# Overview of paratransgenesis as a strategy to control pathogen transmission by insect vectors

**DOI:** 10.1186/s13071-021-05132-3

**Published:** 2022-03-31

**Authors:** Norman A. Ratcliffe, João P. Furtado Pacheco, Paul Dyson, Helena Carla Castro, Marcelo S. Gonzalez, Patricia Azambuja, Cicero B. Mello

**Affiliations:** 1 Programa de Pós-Graduação em Ciências e Biotecnologia, Instituto de Biologia (EGB), Universidade Federal Fluminense (UFF), Niterói, Brazil; 2 Department of Biosciences, Swansea University, Singleton Park, Swansea, UK; 3 Laboratório de Biologia de Insetos, Instituto de Biologia (EGB), Universidade Federal Fluminense (UFF), Niterói, Brazil; 4 Institute of Life Science, Medical School, Swansea University, Singleton Park, Swansea, UK

**Keywords:** Paratrangenesis, Microbiome, Insect vectors, Mosquitoes, Triatomines, Tsetse flies, Sandflies, Environmental safety, Pest control

## Abstract

This article presents an overview of paratransgenesis as a strategy to control pathogen transmission by insect vectors. It first briefly summarises some of the disease-causing pathogens vectored by insects and emphasises the need for innovative control methods to counter the threat of resistance by both the vector insect to pesticides and the pathogens to therapeutic drugs. Subsequently, the state of art of paratransgenesis is described, which is a particularly ingenious method currently under development in many important vector insects that could provide an additional powerful tool for use in integrated pest control programmes. The requirements and recent advances of the paratransgenesis technique are detailed and an overview is given of the microorganisms selected for genetic modification, the effector molecules to be expressed and the environmental spread of the transgenic bacteria into wild insect populations. The results of experimental models of paratransgenesis developed with triatomines, mosquitoes, sandflies and tsetse flies are analysed. Finally, the regulatory and safety rules to be satisfied for the successful environmental release of the genetically engineered organisms produced in paratransgenesis are considered.

**Graphical Abstract:**

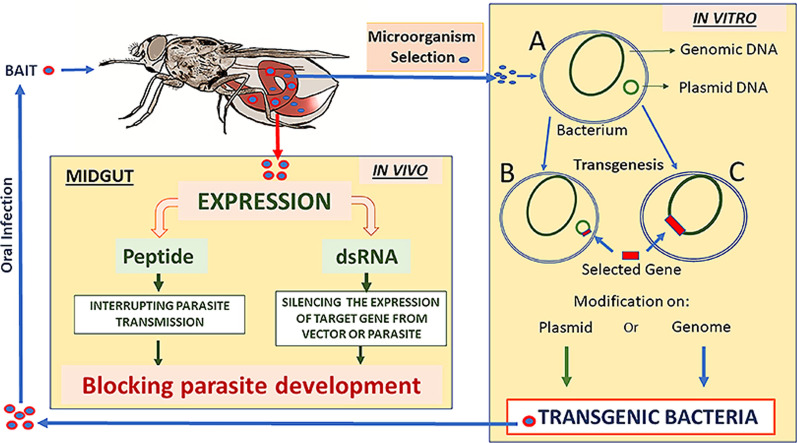

## Background

When the transmission of pathogens by insect vectors is being considered, members of the orders Diptera and Hemiptera deserve particular attention. Many species belonging to these orders are of medical importance, transmitting a great variety of parasites causing diseases, including malaria, Chagas disease, leishmaniasis, sleeping sickness, filariasis, onchocerciasis and arboviruses.

Dipterans comprise approximately 150,000 known species catagorised in about 10,000 genera and 188 families. They include the mosquito (Culicidae), house fly (Muscidae), blow fly (Calliphoridae), robber fly (Asilidae), horse fly (Tabanidae), black fly (Simuliidae), sand fly (Phlebotominae), and gnat (e.g. Sciaridae) [1, 2]. Some species of mosquitoes, i.e. those belonging to the genera *Anopheles*, *Aedes* and *Culex*, act as vectors of many microorganisms that are etiologic agents of diseases, such as malaria, African typanosomiases, yellow fever, dengue, zika, chikungunya, West Nile fever and many others [3–8]. Mosquitoes alone are responsible for as many as 1 million deaths annually, including those from malaria for which high death rates have been occurring for many decades, with poor children aged < 5 years particularly affected [9]. Dengue fever transmitted by *Aedes* mosquitoes is the most common viral disease affecting 3.9 billion people annually in 129 countries, killing approximately 40,000 people every year [9, 10]. In addition, sand flies (genera *Lutzomyia* and *Phlebotomus*) are vectors of *Leishmania* and transmit leishmaniasis in Europe, northern Africa, the Middle East, Asia and parts of South America [11, 12]. Leishmaniasis, as well as onchcerciasis and filariasis transmitted by black flies and mosquito vectors, respectively, also cause permanent disfigurement in those infected. In Africa, several species of tsetse flies (*Glossina* spp.) are vectors of *Trypanosoma brucei rhodesiense* and *T. b. gambiense*, both of which cause sleeping sickness in humans, but these pathogens are not confined to humans since *T. b. brucei*, *T. congolense*, *T. vivax*, *T. evansi*, and *T. equiperdum* also result in African trypanosomiasis in cattle [13, 14]. The economic burden and human suffering caused by these diseases are enormous; for example, the direct costs (illness treatment and death) of malaria alone is estimated to be over 12 billion U.S. dollars per year [15].

Hemipterans have much lower impact than dipterans in terms of the numbers and disease burden of the human parasites vectored. The subfamily Triatominae includes *Rhodnius prolixus* and *Triatoma infestans* that transmit the flagellate protozoan *Trypanosoma cruzi*, which is the causative agent of Chagas disease, throughout South and Central America as well as the USA. The pathology of this disease is horrendous in patients with chronic inflammation of the heart, colon and nervous system. About 6 million people are estimated to be infected with *T. cruzi* in Latin America, of which one third will die from the disease [16]. The hemipterans also include the family Cimicidae, containing the bedbugs, such as *Cimex lectularius*, which also have the potential to transmit human diseases [17]. However, it should not be forgotten that the majority of hemipterans feed on plants and comprise the aphids, white flies and leaf hoppers that are significant vectors of viral diseases of crops [18].

The effect of global warming, the destruction of natural habitats and increases in international travel and trade have all served to increase both the spread of insect vector-borne parasitic diseases and the emergence of new microbial threats of pandemic proportions [19, 20]. For example, *Aedes albopictus*, the highly invasive Asian tiger mosquito, was probably introduced into Europe in 1990 via Italy in imported vehicle tyres. A favourable climate and global warming enhanced the mosquito’s spread and it has vectored outbreaks of chickungunya and dengue brought to Europe by international travellers [19]. The speed by which emerging pathogens can spread may be explosive, as illustrated with the Zika virus pandemic in the Americas; the virus was introduced into Brazil in 2015 and by 2016 it had infected approximately 211,700 people [21]. 

## Challenges and ingenuity in controlling insect-borne diseases

Clearly, with the increased spread of many insect-vectored parasites and the arrival of newly emerging diseases, the need for effective control is absolutely vital. Control methods generally use several strategies and are focussed either on preventing the vector from feeding upon the host and transmitting the disease, or on treating infected individuals with drugs.

There are numerous vector-targeted control techniques, ranging from draining aquatic habitats and removing small domestic bodies of water to the use of mosquito nets, biological control agents, traps, spatial repellants, indoor residual spraying and anti-mosquito bands and creams [22–25]. These are often mediated through an integrated pest management scheme defined by the U.S. Department of Agriculture as “a sustainable, science-based, decision-making process that combines biological, cultural, physical, and chemical tools” [26].

One of the most successful twentieth century methods of controlling insect vectors, however, was the widespread use of insecticides. Among these, dichlorodiphenyltrichloroethane (DDT) was commonly used in the 1950s and 1960s to control mosquitoes and other pest species; however, it was banned in 1972 [27] despite probably saving hundreds of millions of lives [28]. In addition, insects have now developed resistance to the main chemicals used for pest control, namely the organochlorines, pyrethroids, organophosphates and carbamates [29, 30]. This resistance in African *Anopheles* mosquitoes has been described as “a worsening situation that needs urgent attention to maintain malaria control” [31]. Other vector control strategies have been utilised, such as insecticide-impregnated bed nets and microbial control agents [23], but both of these strategies have limitations. Bed nets are of limited use against *Culex* and *Aedes* spp. which bite more often outdoors in the daytime than at night [32]. In addition, the adoption of biological control agents, recognised as non-toxic alternatives to chemicals, may face resistance because of environmental concerns [33]. More recently, ingenious attempts to control insect vectors have turned to genetic modification so that the vector competence to transmit pathogens is reduced or, alternatively, the insect is engineered with a lethal transgene causing death during development [32]. There are, however, issues to be resolved before genetically modified vector insects can be widely released in the field to replace the wild populations, including concerns on the stability of the transgene, fitness of the transformed insects in the field and identification of genes driving favourable traits upon release, as well as ecological concerns over the release of genetically modified (GM) organisms [34, 35].

Once infection has occurred by the insect vector feeding on the human host, then tools are also available to suppress or kill the pathogen. These tools include vaccines and chemotherapy against the invasive parasites. Rapid progress is being made in both of these approaches, with researchers taking advantage, for example, of new information accruing from work on the immune interactions of host and parasite that has revealed key molecules as potential targets for vaccines and drugs [36]. Vaccines that are at present being tested in various phases against malaria [37] dengue [38], Zika [39], chikungunya [40] and leishmaniasis [40] will hopefully soon be available to prevent such infections. At present, the only vaccines for vector-borne diseases on the World Health Organisation-approved list without specific limitations are those against the yellow fever and Japanese encephalitis viruses [40]. In addition, a recent report has shown success rates of 74–77% with the malaria R21/MM vaccine in vaccinated children in Burkina Faso, even after 1 year [41]. The European Medicines Agency also recently accepted Japan’s leading drugmaker, Takeda, filing packages for its TAK-003 dengue vaccine candidate against any dengue virus serotype in people aged 4 to 60 years [42]. Many of these vaccines are therefore already showing favourable results although safety is still of some concern. At present, the prime method for treating protozoan parasites, including *Plasmodium*, *Trypanosoma*, *Leishmania*, *Toxoplasma* and *Entameoba*, is drug therapy, although resistance to these drugs is a growing problem [43–45]. One example of drug resistance is that to the sesquiterpine lactone, artemisinin, and its derivatives; these drugs are used against malaria as they act rapidly to clear parasites from the blood. Artemisinin is derived from the plant *Artemisia annua* and has been used for centuries in traditional Chinese Medicine to treat fevers; since 1980 it has saved the lives of millions of malaria patients [43]. Resistance to artemisinin-derived combinations was first detected in 2008 and has rapidly spread in Southeast Asia. The problem of drug resistance to one drug is exacerbated sometimes by the development of cross-resistance to other drugs. At present, *Plasmodium falciparum*, *P. vivax* and *P. malariae* are showing widespread resistance to a variety of drugs [43, 45]. Similar accounts of drug resistance are reported for leishmaniasis and African trypanosomiasis [43].

Drugs are also available for treating filariasis and onchocerciasis, and although these can be very effective in reducing worm loads, mass drug administration is required [46]. In addition, there is a lack of approved antivirals against the many present and emerging arboviruses [47] and other zoonotic viruses.

In summary, vaccines are still not widely available for preventing vector-borne pathogen infections, and disease vectors are becoming increasingly resistant to pesticides. Furthermore, parasites infecting patients are developing enhanced resistance against therapeutic drugs, and approved drugs are currently unavailable for treating arboviruses that are emerging more frequently and increasingly forming widespread epidemics. Therefore, new drugs and innovative strategies are urgently required for combating these vector-borne diseases [25, 36].

## Definition and advantages of paratransgenesis

**Fig. 1:**
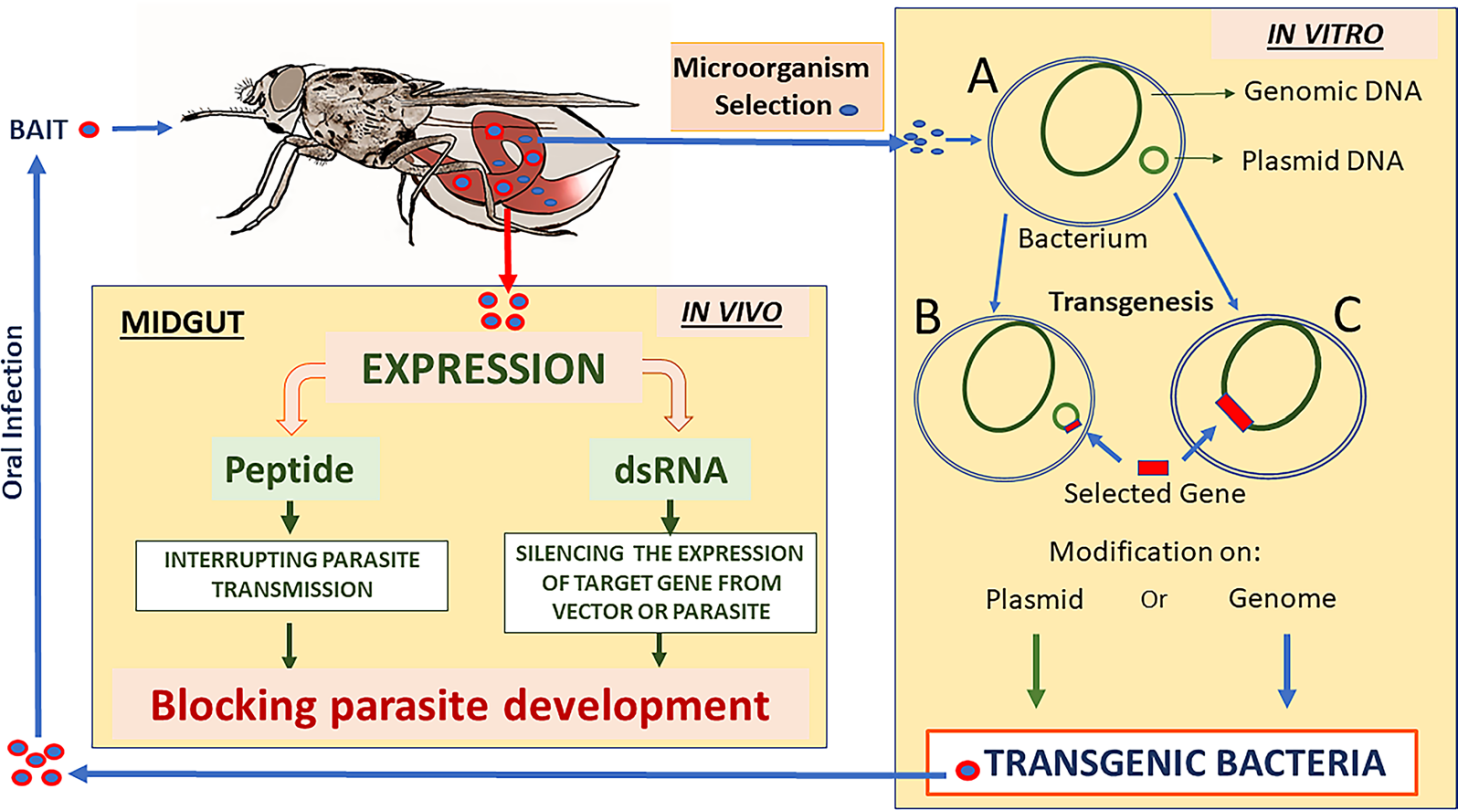
Summary of the analysis and selection of bacteria from vector microbiota for cultivation and genetic modification in vitro. The microorganism (*A*) is genetically modified by the insertion of an exogenous gene in a plasmid (*B*) or directly into the bacterial chromosome (*C*). The transgenic bacteria are offered to adult insects through an attractant bait. In the insect's digestive tract, the genetically modified microorganism expresses a peptide capable of interrupting the transmission of the parasite or a dsRNA that can silence genes in the parasite or the vector, if these are sensitive to RNA interference, thereby blocking parasite development. Abbreviations: dsRNA, Double-stranded RNA

Paratransgenesis is a promising and particularly ingenious strategy currently being developed for controlling vector-transmitted diseases (Fig. 1). It utilises the genetically manipulated native microbiome (mutalistic symbiotic and commensal bacteria, fungi and viruses) [48] of the vector insect to inhibit or kill the disease pathogen. Native symbionts or commensals isolated from the vector are genetically transformed in vitro to produce anti-pathogen factors and then reintroduced to the insect to interrupt the life-cycle of the disease organism [32, 35, 49–51].

The great advantage of this method over genetically transformed mosquitoes is that the transformed bacteria/fungi/viruses used may have the ability to colonise a range of different insect vector strains and even species. In contrast, with transgenic mosquitoes each strain or species may have to be transformed to prevent disease transmission. In addition, it is much easier to produce large numbers of transformed microbes than to generate sufficient numbers of transgenic mosquitoes [34]. Furthermore, the transformed microbe usually undergoes massive multiplication in the target insect vector [52] and may be passed horizontally as well as vertically from one generation to another [53].

A technique similar to—but not true—paratransgenesis involves the transfection of non-native, non-genetically transformed bacteria (or other microbes) to modify the microbiome in the insect gut, thereby altering host physiology and reducing vectoring ability. There are many examples of this process, such as *Serratia marcescens*-blocking *Leishmania braziliensis*, *Trypanosoma cruzi* and *Plasmodium berghei* in their vector insects [32, 54]. One example studied in some detail is *Wolbachia pipientis* (bacteria; family Rickettsiaceae) whose strains widely occur as intracellular pathogens in arthropods and nematodes and which can be inherited transovarially to spread rapidly through insect populations. *Wolbachia* infections in insects can cause incompatibility of the egg and sperm, leading to sterility, feminisation, parthenogenesis and male killing [50], and these outcomes can be used to control vectors. Thus, transfection of the *Wolbachia* wMelPop-CLA strain into *Aedes aegypti* reduces the life-span of the mosquito and its ability to vector chikungunya and dengue [51]. Inhibition of the parasite life-cycle can be achieved even when the mosquito strain is not naturally infected with *Wolbachia.* This inhibitory effect, however, is not universal since *Aedes albopictus* naturally infected with *Wolbachia* still effectively vectors chikungunya [55]. The difficulty in characterising the action of *Wolbachia* as paratransgenesis arises since the strains used may or may not be native to the vector insect, and only untransformed forms of *Wolbachia* are available to inhibit the life-cycles of parasites in different vector insects. *Wolbachia* is an intracellulatr endosymbiont, and to date genetic transformation has not been achieved and in vitro culture is only possible in a few cell lines [56–58]. Many recent articles and reviews on *Wolbachia* are available [59–65]; therefore, the present review is limited to paratransgenesis in which native microbes are transformed and transfected into vector insects.

## Development, requirements and recent advances of the paratransgenesis technique

### Development of paratransgenesis

Paratransgenesis was originally developed in the triatomine, *Rhodnius prolixus*, by Beard, Durvasula and colleagues [66–73] in an attempt to control the transmission of the protozoan parasite, *Trypanosoma cruzi*, the agent responsible for Chagas disease. This pioneering work provided a model for paratransgenesis research in other vector insects that transmit diseases not only to animals and humans but also to agricultural plants [74]. Briefly, the hindgut of *R. prolixus* contains very high concentrations of a Gram-positive, actinomycete bacterium, *Rhodococcus rhodnii.* Upon emergence of the *R. prolixus* nymphs, this organism is acquired in the first instars by coprophagy from the faeces of other members of the colony and is necessary for development of the insects to adults. The bacteria are well-placed to interact with *T. cruzi* as this parasite spends the last stages of its life-cycle in the hindgut of *R. prolixus* surrounded by *R. rhodnii.* The newly emerged nymphs are asymbiotic and can be maintained under sterile conditions and fed transformed or wild-type *R. rhodnii* in a blood meal using an artificial membrane feeder [70]. In the initial studies, the *R. rhodnii* were transformed with an *Escherichia coli*/*R. rhodnii* shuttle plasmid containing antibiotic resistance marker genes [66]; in later studies, however, more stable L1 mycobacteriophage integrative plasmids were used [75]. After the first instar *R. prolixus* are fed with transformed bacteria, they develop to sexual maturity at a rate similar to that of the controls fed with untransformed bacteria. In addition, transformed *R. rhodnii* could be detected in the gut for the 6.5 months of the experiment and also following successive moults [66, 70], thereby demonstrating that a transgenic symbiont could be introduced into a vector insect with no apparent cost to fitness or survival. Subsequently, a gene fragment for the trypanocidal, immune peptide, Cecropin A, from insects was inserted into the *R. rhodnii* symbionts and then aposymbiotic *R. prolixus* were colonised with these transformants or with wild-type control bacteria. Challenge of these two groups of *R. prolixus* fourth instars with *T. cruzi* resulted in 100% and 35% infection rates, respectively, for the control and experimental insects [69, 71]. The 35% of infected *R. prolixus* in the experimental group contained significantly reduced numbers of the final metacyclic forms of *T. cruzi*. These results provided both an innovative method for use in integrated pest management (IPM) programmes for controlling the triatomine vectors of Chagas disease in South and Central America, as well as a stimulus for developing this ingenious technique in other insect vector/parasite associations (Fig. 1).

The results of subsequent paratransgenesis research with other vector insects, including triatomines, are detailed in section Paratransgenesis in different groups of vectors of this review. However, some preliminary discussion on the requirements of and recent advances in the use of paratransgenesis are presented here to facilitate the successful employment of this technique.

### Requirements for successful paratransgenesis

Requirements for successful paratransgenesis include:

i. A culturable, symbiont or commensal bacterium (fungus or virus is occasionally used) should be present in the insect vector, be susceptible to genetic manipulation [76–78] and occupy the same body tissues as the pathogen in the host.
ii. The microorganism ideally should colonise all instars during insect development throughout the life-cycle from first instars into adults. Most bacteria are lost during metamorphosis from larvae to adults, especially in mosquitoes, so that transstadial species such as *Asaia* in mosquitoes are ideal [79].
iii. The microorganism should be non-pathogenic to humans and animals and capable of colonising a range of strains/species of mosquitoes or sand flies, etc. [34].
iv. The ‘fitness’ of the genetically modified microorganism must not be compromised and its stability and normal functioning should be retained within the host vector [70].
v. One or more effector molecules must be identified and then secreted by the recombinant microorganism to have the expected inhibitory effect on the parasite/insect vector interaction. The molecule must have no fitness cost to the insect vector [34, 80, 81].
vi. There must be a way to facilitate the introduction and dispersal of the recombinant microorganism into wild vector insects under field conditions. Initial successes with semi-field trials have been reported in controlling triatomines with transformed *R. rhodnii* and mosquitoes with *Asaia* strains [53, 82].
vii. Approval for use of the paratransgenesis technique from regulatory bodies and local populations must be sought. There are serious safety concerns about the release of genetically modified organisms in the field which will need to be addressed by environmental risk assessments. The risks of horizontal genetic transmission to the genomes of other organisms must also be minimised [34, 83].

### Technical advances in the use of paratrangenesis

Most important innovative methods undergo improvements to increase efficiency and optimise the outcomes. This is most certainly true for paratransgenesis, with advances made in most stages, as outlined in the following sections, and shown in Table 1.

**Table 1:** Summary in approximate chronological order of some of the important advances in the development of paratransgenesis in vector insects

Insect vectors	Transformed microbes used + effector genes	Paratransgenesis innovation	References
*Rhodnius prolixus*	*Rhodococcus rhodnii* + thiostrepton resistance	Original technique described	[66]
*R. prolixus*	*R. rhodnii* + cecropin A	Killing of *Trypanosoma cruzi*	[69, 71]
*R. prolixus*/*Triatoma infestans*	*R. rhodnii*/*Corynebacterium* sp. + AMPs, rDB3 and endoglucanase	Combinations of effector molecules kill *T. cruzi*	[77, 82]
*Anopheles gambiae*	*Metarhizium anisopliae* + scorpine and scorpine fusion protein	Combinations of effector molecules kill *Plasmodium*	[100]
*Anopheles stephensi*	*Serratia* ASI + 5 anti-*Plasmodium* effector proteins	Combinations of effector molecules kill *Plasmodium*	[120]
*Anopheles gambiae*	Microbiome endosymbionts fully identified for the first time	High-throughput sequencing introduced	[132]
*R. prolixus*	*R. rhodnii* + rDB3 antibody fragment	Semi-field simulation of transgenic bacteria spread in CRUZIGARD	[73]
*Homalodisca vitripennis*^a^	*Pantoea agglomerans*^gfp^	Semi-field simulation of transgenic bacteria spread in hydrogel	[128]
*An. stephensi/An. gambiae*	*Asaia*^gfp^	Semi-field simulation of transgenic bacteria spread	[53]
*An. stephensi*	*Serratia* AS1^-mCherry^ and AS1^-gfp^	Semi-field simulation of transgenic bacteria spread	[120]
*R. prolixus*	*R. rhodnii* and *Gordona rubropertinctus*	Model showing negligible risk of horizontal transfer of transgenic bacteria	[133]
*An. stephensi*	*Serratia* ASI^-gfp + mCheery^ and kanR genes/+ microbiome in vivo	No horizontal transfer of transgenic bacteria genetic material in vivo	[134, 162]
*Anopheles* spp.	*P. agglomerans*	Modelling paratransgensisi^b^	[130]
*An. stephensi*	*Serratia* ASI^-gfp + mCheery^ and kanR genes/+ microbiome in vivo	Transiently expressed plasmids for checking environmental safety of released genes	[134]
*An. stephensi*	*Asaia* + scorpine	Transgene only expressed after blood meal, thus reducing fitness costs	[126]
*R. prolixus*	*R. rhodnii and Escherichia coli* expressing dsRNA	RNAi and knockdown of vector genes^c^	[74, 111]
*Anopheles* spp.	*Asaia* RNaseIII mutant created	Potential for developing an efficient RNAi-based paratransgenesis for vector or parasite gene knockdown	[110, 115]
*An. gambiae*	CRISPR/Cas9 is a new method of microbe transformation	Potential to transform microbes for paratransgenesis and also mediate gene silencing	[102, 135]
*Aedes albopictus*	MDVs	miRNA expression system with recombinant MDVs stable for silencing mosquito genes	[90]

*dsRNA* Double-stranded RNA, *gfp*   green fluorescent protein,* kanR* kanamycin resistant,* mCherry *red fluorescent protein,* MDVs *mosquito densoviruses,* miRNA* microRNA,* rDB3* antibody fragments (encoding murine VH/K which binds progesterone),* RNAi * RNA interference

^a^The glassy-winged sharpshooter, a hemipteran like the triatomines

^b^See also the paratransgenesis modelling paper by Li et al. [136] based on systems of differential equations

^c^This will lead to RNA interference-based paratransgenesis; see, for example, Asgari et al. [115]

#### Analysis of microbiomes

Advances in molecular techniques beyond the* 16S* RNA gene method for the analysis of vector insect microbiomes have been made with, for example, high-throughput sequencing (HTS) commonly used for the complete analysis of all microbes in a sample [83]. The* 16S* RNA method uses just one gene for analysis while HTS fragments all the DNA in a sample, sequences these and then fits them together for analysis [84]. Thus, with HTS all groups of microorganisms in insect tissue samples can be identified; those selected for paratransgenesis must then be amenable to multiplication with traditional culture techniques. In addition, culturomics has recently been successfully introduced to identify previously unknown bacterial species in the vector gut microbiome. Basically, culturomics consists of multiple culture conditions combined with matrix-assisted laser desorption/ionisation–time of flight (MALDI-TOF) mass spectrometry or* 16S* ribosomal DNA (rDNA) amplification and sequencing [50, 85].

The choice of symbiotic microorganisms as recombinant candidates for the expression of effector molecules in paratransgenesis has also been extended from bacteria to include viruses and fungi, although the majority of studies have utilised bacterial symbionts [70, 83, 86]. Novel viruses and fungal genera have been identified in *Culex pipiens* by shotgun metagenomic sequencing, which is a HTS, PCR-independent technique, as well as by culture-dependent methods [87]. Wild mosquitoes are also commonly infected with insect-specific viruses belonging to several families, including the Densovirinae and Flaviviridae [86, 88], and these appear to suppress arbovirus infections in mosquitoes by superinfection suppression [51]. Work with mosquito densoviruses (MDVs) has demonstrated their potential use in paratransgenesis. MDVs are environmentally stable and colonise natural mosquito populations by vertical and horizontal transmission, and their host specificity is restricted to mosquitoes [89, 90]. They also have small genomes that are easily modified genetically to express foreign effector genes with potential for the transformation of target vector insects [90–93]. The recombinant viruses initially produced, however, were apparently replication defective and unable to undego secondary transmission due to the loss of viral capsid proteins essential for replication [90]. This problem has now been overcome using a microRNA (miRNA) expression system in which the recombinant MDVs are stable and self-replicating and induce silencing of mosquito genes [90]. In addition, the problem of large-scale production of the recombinant MDVs has been solved, allowing field testing experiments [94].

Fungi with long environmental survival times as spores and the ability to infect insects directly through the exoskleleton also have the potential for use in paratransgenesis. Mosquitoes, triatomines and sand flies have been shown to have extensive mycobiomes, although current knowledge of the interaction of these mycobiomes with vector insects and their infecting pathogen associations is very limited [83, 95–97]. Yeast species, such as *Wickerhamomyces anomalus*, have been isolated from both laboratory and wild colonies of *Anopheles* and are widespread in adult mosquito tissues, suggesting possible use in paratransgenesis [81, 98]. This potential has been confirmed with genetically modified yeast delivering double-stranded RNA (dsRNA) to a *Drosophila* sp. pest of soft fruits which, following ingestion, resulted in decreased locomotor activity and reduced egg-laying of the adult insects [99]. Another significant study was conducted on the entomopathogen, *Metarhizium anisopliae*, which was transformed to express anti-*Plasmodium falciparum* molecules, including various combinations of scorpion toxin (scorpine), artificial salivary gland and midgut (SMI) molecules and PfNPNA sporozoite-binding antibody. Transgenic *Anopheles* infected with *M. anispliae* expressing a combination of scorpine with SMI peptides resulted in > 98% inhibition of sporozoite levels in the salivary glands [100]. Subsequent to these studies, however, little progress seems to have been made in the use of fungi in paratransgenesis for the control of pathogen transmission by vector insects. This is surprising since entomopathogenic fungi can easily be mass produced and genetically transformed, do not infect vertebrates and have previously been used in the field to control insect pests. There would also be a synergistic effect since both the mosquito and the malarial parasite would be inhibited by the transformed fungus [101].

#### Transformation of symbionts

Once the symbionts have been selected, they are transformed to carry effector genes to inhibit the life-cycle of the pathogen in the vector insect or even in the vector itself. Various plasmid vector systems are usually used for this genetic transformation. For example, in studies of paratransgenesis in the vector insect, *Rhodnius prolixus*, L1 mycobacteriophage integrative plasmids inserted genes into the genome of the symbiotic bacterium, *Rhodococcus rhodnii*, to give highly stable constructs [77] One recent development has been the introduction of CRISPR-Cas (Clustered Regularly Interspaced Short Palindromic Repeats [CRISPR] with CRISPR-associated [Cas] endonuclease or enzyme) genome editing systems to transform the genomes of the insect gut microbiome. Not only can such systems transform bacteria by introducing one or more specific genes but it can also mediate gene silencing [102–104]. CRISPRs are derived from prokaryotes and include an endonuclease (Cas9) guided by a guide RNA (gRNA) to cut the chromosome at a specific site [32]. One advantage of this system is that the integration of the transgene is not only site-specific but also highly stable. The CRISPR technique has been applied successfully to edit an outer membrane gene (*ompA*) of a gut symbiont of *Aedes aegypti* to determine its role in biofilm formation in the vector gut [105], as well to transform several mosquito species to alter and drive specific genes into different generations [32, 106, 107]. The CRISPR system can transform microbes for paratransgenesis and also mediates gene silencing, resulting in more consistent and robust knockdowns with fewer off-target events than RNA interference (RNAi; see detailed comparative review on CRISPR and RNAi in [108]). RNAi knockdown techniques, however, have been invaluable in mosquitoes to investigate immune gene functions in relation to parasite interactions [83, 109]. In earlier studies, RNAi knockdown in *Plasmodium* was hampered by an apparent lack of appropriate RNAi machinery, but it is now possible by engineering two components, Argonaute 2 and a modified short hairpin RNA, into the parasite. These transgenic parasite lines, although not immediately transferable to the field, will be invaluable for studying *Plasmodium* gene function [110]. Thus, with both RNAi and CRISPR-Cas systems, it should be possible for multiple genes to be inhibited simultaneously and so prevent parasites developing resistance. The next step will be to inhibit parasite development within the insect vector using these techniques [83].

In addition, a modified form of paratransgenesis, termed RNAi-based paratransgenesis, has recently been introduced in which the transformed microbes deliver dsRNA instead of the usual effector proteins [74, 111–116] (Fig. 1). This method of delivery is ingenious as previously it was necessary to either inject or feed the target insects with the dsRNA, with both of these methods having significant disadvantages [117]. Injection is labour intensive, often kills many insects and induces immune/stress responses to potentially confuse interpretation of results, and the RNAi effect may be transient in long-lived species. Likewise, with feeding insects the dsRNA, the effect may be temporary and require repeating several times [74], although prolonged knockdown of the *TsetseEP* gene was achieved in *Glossina* by feeding dsRNA [118]. Using bacteria to express dsRNA has been successfully employed in the haematophagous triatomine, *R. prolixus* [74, 111], and the phytophagous crop pest, *Frankliniella occidentalis* [74]. In *R. prolixus*, an RNaseIII-deficient endosymbiont strain of *Rhodococcus rhodnii* had dsRNA expression cassettes stably incorporated into the chromosome and was used to successfully knock down *Nitrophorin-1* and *Nitrophorin-2*. The knockdown phenotype produced colourless salivary glands in contrast to the cherry-red glands of the controls [74]. Likewise, a dsRNA expression cassette for *Vitellin*, responsible for producing approximately 80% of oocyte protein in *R. prolixus*, resulted in a significant 72.3% reduction in first instar-eclosed insects per adult insect per day. The transformed *R. rhodnii* not only persisted > 250 days in the *R. prolixus* gut with no apparent effects on insect fitness, but was also horizontally transmitted by coprophagy [74]. Also in *R. prolixus*, using *E. coli* expressing dsRNA, genes *RHBP* (*Rhodnius* heme binding protein) and *CAT* (catalase), involved in antioxidant activity and oocyte development were knocked down between 65 and 96%, respectively [111]. Finally, in the thrip, *Frankliniella occidentalis*, a similar strategy was adopted, except that alpha tubulin was targeted (*tubulin alpha1*), resulting in depletion of *tubulin* mRNA levels and significantly increased mortality of adults [74]. These studies indicate the real possibility of using symbiont-mediated RNAi to control both vectors of disease and agricultural pests. This technique has now been developed to mediate honey bee physiology and kill parasitic *Varoa* mites [116], and is being advanced to produce a mutant *Asaia* strain for RNAi-based paratransgemesis in *Anopheles* [115]. Future studies should be aware that using RNaseIII mutant bacteria has been shown to improve the delivery efficiency of dsRNA compared with normal transformed bacteria still producing RNAseIII [119].

#### Choice of effector molecules

The original inspirational studies on paratransgenisis in *R. prolixus* used the native endosymbiont, *R. rhodnii*, to deliver either a functional antibody fraction or Cecropin A, an insect antimicrobial peptide, as effector molecules against *T. cruzi* [66, 71]. Subsequently, numerous other effector proteins have been identified and used in paratransgenesis, with the majority associated with mosquitoes and against *Plasmodium* spp. (for more details see [81, 120–123] and section Mosquito microbiomes: native endosymbiotic bacteria in paratransgenesis of present article). In addition, a number of advances in use of effectors have been made. First, antimicrobial peptides are frequently used in paratransgenesis against various stages of *Plasmodium*; in order to facilitate this, Carter et al. [124] tested a range of 33 such molecules. These peptides were fed to *Plasmodium*-infected anopheline mosquitoes in the first 24 h of the sporogonic stage. Analysis identified seven peptides, mainly from bee and wasp venoms, that mediated significant killing of the parasites and had limited effects on mosquito fitness in terms of fecundity and longevity. It should be noted that this study involved feeding the peptides directly to infected insects rather than through secretion by transformed native symbionts. Second, studies have found that combinations of effector molecules are much more effective at killing parasites than single molecules (e.g. [75, 100, 120, 121, 124, 125]). Most of these studies, however, were with parasites mixed with effector proteins in vitro or with transgenic mosquitoes expressing combinations of proteins rather than by paratransgenesis with multiple effectors secreted by a single transformed symbiont. The outstanding paratransgenesis research of Fang et al. [100] and Wang et al. [120] with *Anopheles gambiae* and *An. stephensi*, respectively, however, describes the multiple simultaneous expressions of effector molecules by microbes. In *An. gambiae*, *Metarhizium anisopliae* was transformed to deliver scorpine as well as an [SM1]_8_:scorpine fusion protein, resulting in a 98% reduction of *Plasmodium falciparum* sporozoite counts [100]. Also, in *An. stephensi*, a *Serratia* strain of symbiotic bacteria (*Serratia* ASI) was discovered, capable of simultaneously expressing five anti-*Plamodium* effector proteins [120] (see also *Pantoea agglomerans* and *Serratia* in section Mosquito microbiomes: native endosymbiotic bacteria in paratransgenesis of present article). These combinations of proteins from transformed *Serratia* ASI or *M. anisopliae* were more effective at reducing parasite oocyst or sporozoite numbers, respectively, than those from symbionts producing just a single effector molecule [100, 120]. This is an important step forward since such combinations of anti-parasite effector molecules with various modes of action can be optimal for preventing the development of resistance by parasites. Third, one significant problem with transgenic symbionts released in the field is their potential loss of ability to compete with the microbiome already established in the gut of wild vectors [126]. For example, *Asaia bogorensis* colonises a range of vectors, including *Ae. aegypti*, *Ae. albopictus* and *An. stephensi*, and has been engineered to produce anti-*Plasmodium* effectors. Shane et al. [126] reasoned, however, that genetic modification of the bacteria may lead to a significant loss of fitness as a cost of the prodution of the effector protein, leading to lack of competiveness when released in the field. This problem was resolved by isolating blood meal-induced promotors (BMI) activated only during vector feeding on blood and exposure to nutrients [126]. Plasmids expressing the anti-*Plasmodium* protein scorpine under the control of the BMI promoters were constructed and transferred into *Asaia* sp. *SF2.1* strain by electroporation. The *Asaia* BMI strains, in comparison to the constitutive scorpine-expressing control strain, had significantly increased maximum growth rates, enhamced ability to compete when co-cultured with wild-type *Asaia* and increased colonisation of mosquito midguts. The BMI strains also resulted in a significant reduction in oocyst numbers compared with the constitutive scorpine-producing control [126]. The authors hypthesised that for release in the field more than one effector protein should be expressed by the transformed symbiont to reduce chances of resistance. In addition, for the sake of stability, the effector genes should be inserted into the *Asaia* chromosome rather than carried on a plasmid (see CRISPR in section Transformation of symbionts of present article).

#### Tranfection into insect vectors

**Fig. 2:**
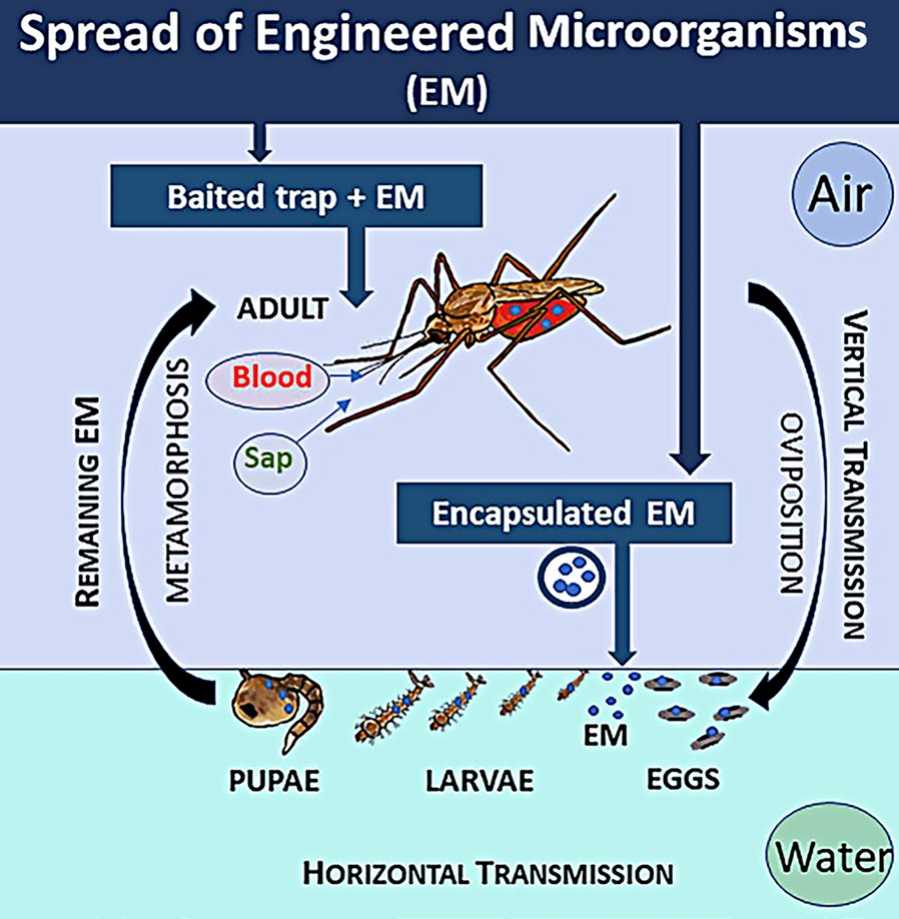
Spread of engineered microorganisms (EM) into wild mosquitoes. EM can be directly offered to winged adults through a baited trap or encapsulated and seeded or oviposited into water to contaminate aquatic juvenile forms. Females containing EM can also contaminate eggs laid on land, enabling vertical transmission. The selected bacterial species should preferably remain in the different stages of mosquito development or even be transmitted horizontally within this host. In this way, EM can remain permanently and cyclically in the environment

A significant problem for paratransgenetic control of disease vectors is the delivery of the transformed symbiont to a specific wild insect vector population under field conditions [127] (Fig. 2).

This process requires rapid spread of the foreign genes through the native population, without either fitness costs to the target vector or the occurrence of transfection of non-target insects. This technique could potentially be incorporated into IPM programmes. but problems associated with the release of GM organisms have yet to be fully resolved (see section Concluding remarks including safety and environmental concerns of present article). There have, however, been attempts to simulate natural conditions and investigate the potential of transfecting vectors in the wild. Some of these studies are more than 10 years old [73], with the major regulatory barriers to GM organisms presumably still not statisfied. The following are more recent pilot experiments more or less simulating natural conditions for transfection.

Mancini et al. [53] used large cages to study the horizontal and vertical transfection of *Asaia* sp.-transformed bacteria expressing green fluorescent protein (GFP), *Asaia*^gfp^, into laboratory-reared *Anopheles stephensi* and *An. gambiae* populations. Transfection occurred either by the release of paratransgenic male mosquitoes or from feeding on cotton pads soaked in sucrose plus 10^8^ transformed *Asaia*^gfp^ bacteria/ml. Transfection was monitored after 5, 12 and 20 days by fluorescence microscopy and PCR. The results showed the efficient horizontal spread of *Asaia*^gfp^ into both *An. stephensi* and *An. gambiae*. For example, in *An*. *stephensi*, the release of paratransgenic males resulted in a 73% infection rate in 400 mosquitoes after 20 days. In addition, experiments on vertical and trans-stadial transmission in *An. gambiae* resulted in 78% of fourth instars and 44% of the newly emerged adults with *Asaia*^gfp^. In conclusion, this semi-field pilot study illustrates the feasibility of transfecting transformed bacteria into populations of mosquitoes [53].

Arora et al. [128] also used a simulated field study to address the problem of transfecting a pest insect, the glassy-winged sharpshooter, *Homalodisca vitripennis* (a hemipteran like the triatomines), with a transformed bacterium, *Pantoea agglomerans*, expressing a GFP. This insect is a vector of *Xylella fastidiosa* which is a bacterial pathogen of grapes and citrus fruits. The engineered *P. agglomerans* were microencapsulated in an alginate hydrogel and after ingestion by field-collected *H. vitripennis*, the bacteria colonised the foregut for up to 15 days. The bacteria were only released from the gel during the flow of plant sap into the foregut of feeding insects. More recently, it has also been shown that *P. agglomerans* can be transmitted horizontally between *H. vitripennis* and therefore may be self-sustaining [128].

Finally, Wang et al. [120] used laboratory cage experiments containing virgin female and male *An. stephensi* mosquitoes fed, respectively, with *Serratia* AS1^−mCherry^ and AS1^-gfp^, to monitor how *Serratia* AS1 colonised and persisted in these mosquitoes. Subsequently, The results showed that all the offspring larvae and adults carried both fluorescent proteins so that the transformed *Serratia* AS1 spread through the whole mosquito life-cycle horizontally, vertically and transstadially. Additional experiments also showed that the bacteria persisted in multiple subsequent generations [120] (for more details, see section Mosquito microbiomes: bacteria of present article).

These studies are important as they describe the successful transfection and persistence of transformed bacteria into insect vector populations as well as the use of microencapsulation of engineered bacteria to limit their release and contamination of the environment (Fig. 2). These are significant steps in the evolution of paratransgenesis from the laboratory to the field [128, 129]. In addition, the results of semi-field trials provide information for formulating models of the efficacy of paratransgenesis and ways of improving the spread of transformed bacteria. For example, information on the distriburion of sugar baits could be used to help prevent a malaria outbreak and influence control policies [130]. On a cautionary note, however, the value of these semi-field pilot studies has been questioned since the insects tested are sometimes inbred laboratory strains and the doses of microbes used extremely high so the results obtained may not reflect the normal responses occurring in nature [131]. When cage trials are undertaken, it is also important to carefully assess the effects of genetic transformation on mosquito fitness in all developmental stages, including percentage of egg hatching. The above advances are summarised in Table 1.

## Paratransgenesis in different groups of vectors

The majority of studies on paratransgenesis have been conducted using symbiotic or commensal bacteria for transformation (see section Analysis of microbiomes of present study). The composition of the bacteria in insects is highly dynamic and varies not only from vector species to species but also according to stage of development, sex, nutrition, habitat, geographical region of the insect and location in the insect [83, 97, 132–141]. The bacteria live in the intracellular or extracellular environment of the insect host and preferentially colonise the midgut and less frequently the salivary glands and reproductive organs [142, 143].

### Mosquito microbiomes

There are over 3567 species of mosquitoes classified into 41 genera [144], but members of just three genera, *Anopheles*, *Aedes* and *Culex*, are responsible for the transmission of the majority of human diseases [9].

Studies on the microbiome composition of mosquitoes are most important since these have revealed the roles of the constituent microbes in the nutrition, physiology, immunity, metabolism, reproduction, longevity and even behavior of the host mosquitoes (e.g. [145–149]). In addition, the microbiome influences the relationship of the vector insect with infecting parasites and pathogens (e.g. [97, 120, 146, 149–158]). An excellent review on the interaction of the mosquito gut microbiota with the immune system and the pathogens is provided by Gabrieli et al. [159] who emphasises the importance of understanding this trilogy in order to maximise control strategies. Therefore, knowledge of the insect vector microbiome is vital for identifying microbes for use in paratransgenesis and for optimising the mass production for release of transgenic mosquitoes to control malarial parasites and arboviruses [34, 51, 83, 120, 160–163].

In the last decade, the realisation of the importance of the mosquito microbiome has resulted in over 300 publications on this topic [164]. This in turn has produced large amounts of data from many species in various physiological states and from different habitats, using alternative sampling and analysis techniques. This problem has made it difficult to compare the research results on mosquito microbiomes from different studies [51, 161, 164, 165]. Dada et al. [164] have therefore created a Mosquito Microbiome Consortium (www.mosquito-microbiome.org) as a repository for rationalisation of these data and to provide guidelines for conducting mosquito microbiome research to enhance collaboration. This Consortium focusses on four areas, namely: (i) sampling/experimental design; (ii) metadata collection; (iii) sample processing and controls; and (iv) data handling and analysis.

It is highly recommended to read this Consortium paper [164] as well as the publications of Romulo and Gendrin [157] and Rodríguez-Ruano et al. [165] for rationalisation of future research efforts and protocols. Consultation of the mosquito microbiome literature does, however, reveal some consistences in the the composition of bacterial species (see following subsections).

#### Mosquito microbiomes: bacteria

Bacteriomes of *Aedes* spp. and *Anopheles* spp. mosquitoes consist primarily of Gram-negative species, with as many as 98 genera described in anophelines [166]. Many of these bacteria are found in the midgut although the salivary glands and reproductive organs are also involved. In *Anopheles culicifacies*, the salivary glands are reported to contain more diverse microbial communities than the gut [142].

A detailed account of the bacteriome distribution in *Aedes* has been given by Scolari et al. [51]. Relatively few taxa, however, usually dominate, and these are often referred to as the core microbiota [156]. These may be highly variable depending upon the host stage and sex, the habitat, as well as whether the mosquitoes were laboratory-reared, field-caught and parasitised or not [149, 167–169],

The mosquito bacteriome is mainly composed of the Gram-negative phyla Proteobacteria and Bacteroidetes, but there are also representatives of the Gram-positive phyla Firmicutes and Actinobacteria. The Proteobacteria contain many species with great potential for paratransgeneisis experiments in mosquitoes and include the genera *Asaia*, *Enterobacter*, *Pantoea*, *Serratia*, *Aerobacter*, *Aeromonas*, *Alicyclobacillus*, *Bacillus*, *Clostridia*, *Elizabethkingia*, *Escherichia*, *Flavobacterium*, *Geotrichum*, *Klebsiella*, *Lactobacillus*, *Micrococcus*, *Proteus*, *Pseudomonas*, *Shewanella*, *Spingomonas*, *Thorsellia* and *Wolbachia* (e.g. [51, 83, 97, 132, 137, 140, 141, 149, 151, 160–163, 168–179]).

This list is incomplete, as shown by Tainchum et al. [179], who recorded five new genera in anophelines from Thailand, and by Nilsson et al. [180] who, working with *Anopheles darlingi* from the Amazon Basin, reported dominance of *Escherichia*/*Shigella*, *Pseudomonas* (all Proteobacteria) and *Staphylococcus* (phylum Firmicutes). Whether there are any differences in the microbiota of *Aedes*, *Anopheles* and *Culex* has also been the subject of study (e.g. [156]). It has been shown in a comparative study of the microbiome of field-collected *Aedes*, *Anopheles* and *Culex* carried out in the USA that there are similarities in the bacteria components in the gut [180]. Ecology seems to be important since different mosquito species from similar environments share core bacteria. The environment determines the nature of the food resources, such as the plants and nectar, as well as the composition of the microbiota at the breeding sites. The microbiota of the larvae will be acquired from the surrounding water, and a fraction will be retained by the adults following moulting (Fig. 2); the remainder will be modified following a blood or sugar meal or even parasitisation and co-occurrence and co-exclusion interactions in the microbiome with, for example, *Wolbachia* [135, 156, 175]. In fact, the mosquito gut has been described as a “selective eco-environment for its microbiome”, favouring enteric bacteria, such as the Enterobacteriaceae, with high redox capacities to manage the oxidative and nitrosative stresses from the digestion of the blood meal [181]. Finally, although genera of bacteria found in laboratory-reared and wild mosquitoes are similar, wild mosquitoes lose component microbiota within one generation of laboratory rearing [161].

#### Mosquito microbiomes: native endosymbiotic bacteria in paratransgenesis

Relatively few native bacterial species (Table 2) from the midgut of mosquitoes have been exploited for developing paratransgenesis for the control of disease and malaria in particular. These include *Asaia*, *Pantoea*, *Serratia*, *Enterobacter*, *Escherichia*, *Chromobacterium* and *Pseudomonas.*

**Table 2:** Genetic manipulation of bacteria, fungi and viruses with potential use in mosquitoes for paratransgenic control of *Plasmodium*

Transformed microbes used + effector genes	Insect vectors	Experimental results with transformed microbes	Key references
*Asaia* BMI strain + scorpine	*An. stephensi*	*Plasmodium berghei* reduction in oocyst numbers in vivo	[126]
*Asaia*^gfp^ *Asaia*^DsRed^ *E. coli*^DsRed^	*An. stephensi*	*P. berghei* ANKA, strain PbGFP_CON_ shows co-localisation of parasites and bacteria	[196]
*Asaia*^gfp^	*An. gambiae*, *An. stephensi*	Semi-field transfection study only	[53]
*Asaia* + scorpine *Asaia* + anti-Pbs21 scFv-Shiva1 immunotoxin	*An. stephensi*	*P. berghei* reduction in oocyst numbers in vivo	[193]
*Asaia*^gfp^	*An. stephensi*	Transfection study only	[190]
*Enterobacter*^gfp^ + defensin	*An. stephensi*	Transfection study only	[218]
*Pantoea* + mPLA2 *Pantoea* + Pro:EPIP *Pantoea* + Shiva1 *Pantoea* + scorpine *Pantoea* + (EPIP)_4_	*An. stephensi*, *An. gambiae*	*P. berghei* *Plasmodium falciparum* Inhibition of 85–98% oocyst formation in vivo	[121]
*Serratia* AS1^-gfp^ *Serratia* ASI^−mCherry^	*An. gambiae*, *An. stephensis*	Semi-field transfection study only	[120]
*Serratia* ASI^-gfp^ + mCherry and kanR genes + microbiome in vivo	*An. stephensi*	No horizontal transfer of transgenic bacteria and transient plasmid expression	[134, 162]
*Serratia* ASI^−mCherry^	*An. stephensi*, *Culex pipiens*, *Cx. quinquefaciatus*, *Cx. theileri*	Transfection studies through different routes	[214]
AgDNV^-gfp^	*An. gambiae*	Transfection of GFP- labelled viruses	[91]
*Metarhizium anisopliae* + [SM1]_8_ *M. anisopliae*-PfNPNA +1 *M. anisopliae* + scorpine *M. anisopliae* + [SM1]_8_:scorpine	*An. gambiae*	*P. falciparum* reduced sporozoite counts	[100]

*AgDNV* *Anopheles gambiae* densonucleosis viruses,* DsRed* discosoma red, mCherry red fluorescent protein, kanR  kanamycin resistant

##### ***Asaia***

*Asaia* commonly infects mosquitoes via plant nectar and has been found in a large range of mosquitoes both from wild and laboratory strains. It is present in many *Anopheles* (*Nyssorhynchus*) species, including the important malaria vectors, *An. stephensi*, *An. gambiae*, *An. fluviatilis* and *An. darling*, as well as in *Aedes aegypti*, *Ae. albopictus* and the *Culex pipiens* complex [51, 163, 173, 182–189].

*Asaia* has great potential for development in paratransgenesis as not only is it widely distributed in mosquitoes but it also colonises the midgut, salivary glands and reproductive organs of both male and females. In addition, *Asaia* is horizontally and vertically transmitted, present in different stages of mosquito development and can be grown in culture and genetically manipulated (e.g. [138, 173, 190–192]). In addition to this potential of *Asaia* in paratransgenesis, wild *Asaia* strains can also inhibit the development of malarial parasites through the production of toxic proteins [183, 193], reduce the malarial parasite load by activation of the basic immune system of *Anopheles* after an infected blood meal [194] and inhibit competing *Wolbachia* infections [195]. Despite the potential of *Asaia* and other symbionts for the paratransgenesis control of malaria, there seems to be more emphasis on producing transgenic mosquitoes for release and control programmes rather than the potentially safer paratransgenesis alternative [10, 34, 122, 125].

Wang and Jacobs-Lorena [122] compiled a comprehensive table of possible anti-*Plasmodium* effector molecules, recognising four classes: (i) parasite killing; (ii) interaction with parasites; (iii) interaction with mosquito midgut or salivary gland epithelia; and (iv) manipulation of mosquito immune system. These molecules, together with those identified by Carter et al. [124], provide a useful choice for delivery by mosquito symbionts such as *Asaia*.

In order to determine more about the *Asaia*–mosquito interactions and test the suitability of *Asaia* for use in paratransgenesis with mosquitoes, Favia et al. [190] undertook a study on the kinetics of infection of *Asaia* in *An. stephensi*. Analysis of infected mosquitoes fed with GFP-tagged *Asaia* (*Asaia*^gfp^) showed that the bacteria colonised the female gut and salivary glands, the same compartments occupied by the malarial parasite during development. In addition, the larval gut and adult male reproductive system were massively invaded. Therefore, *Asaia* could potentially be orally and venerally transmitted, and, as reported previously, passed vertically from mother to offspring [190]. The bacteria remained in the adults throughout their lives and could be transmitted using simple sugar solutions. Capone et al. [196] also reported in *An. stephensi* that *Plasmodium berghei* genetically modified to express GFP (PbGFPcon) induced a host immune response but with no adverse effect against the midgut native population of *Asaia*. In fact, 2 days after PbGFPcon infection, the *Asaia* were enhanced by tenfold in mosquitoes. Other assays using *Asaia*^gfp^ and *Asaia*^DsRed^showed the co-localisation of the bacteria with *Plasmodium berghei* (PbGFPcon) in the salivary glands and midgut, an optimal situation for reducing the vectorial capacity of the mosquito with transformed *Asaia* releasing anti-*Plasmodium* factors [196].

Subsequently, Mancini et al. [53] undertook a semi-field pilot study of the horizontal and vertical transfection of *Asaia*^gfp^ into laboratory-reared *Anopheles stephensi* and *An. gambiae* populations and confirmed the efficient transmission of the bacteria in both species (see details in section Tranfection into insect vectors in present article). Thus, the feasibility of transfecting transformed *Asaia* into populations of mosquitoes was confirmed.

Successful experiments modifying *Asaia* to secrete heterologous proteins into the *An. stephensi* midgut and inhibit *P. berghei* were first reported by Bongio and Lampe [193]. *Asaia bogorensis* were genetically screened, and an efficiently secreted siderophore receptor protein was fused with the antiplasmodial gene scorpine or with an anti-Pbs21 scFv-Shiva1 immunotoxin. These *Asaia* strains were fed to mosquitoes that were then challenged with a *P. berghei*-infected blood meal; 2 weeks later mosquitoes were dissected and oocyst numbers on the midgut counted. Significant reductions in oocyst numbers occurred in both *Asaia* strains compared with the controls (*P* < 0.0001 and < 0.0006, respectively, for scorpine and the immunotoxin transformants) [193]. More recently, however, Shane et al. [126] reasoned that genetic modification of the bacteria may lead to a significant loss of fitness and competiveness in the field. These researchers therefore constructed *Asaia* BMI strains (bacteria with blood meal-induced promotors). These strains, in comparison to the constitutive scorpine-expressing controls, showed significant increases in maximum growth rates, in the ability to compete with wild-type *Asaia*, in the colonisation of mosquito midguts and in the inhibition of oocyst numbers [126] (see details in section Choice of effector molecules of present article).

##### *Pantoea agglomerans* (= *Enterobacter agglomerans*)

*Pantoea agglomeratus* was the most prevalent of 20 genera of symbiotic bacteria reported in wild-caught *An. gambiae* and *An. funestus* mosquitoes from Kenya and Mali [170]. There are also other reports of *P. agglomeratus* in anopheline, *Aedes* and other culicine mosquitoes from around the world [51, 197–199]. In addition, this bacterium is present in laboratory strains of *Anopheles stephensi*, *An. gambiae* and *An. albimanus* [200, 201]. *Pantoea agglomeratus* has been developed for potential paratransgenesis to prevent the transmission of malaria in mosquitoes [81, 114, 202], and of plant diseases and pests in agriculture crops [74, 129, 203] (see section Tranfection into insect vectors of present article). *Pantoea agglomeratus* has great potential for use in paratransgenesis as it naturally infects mosquitoes, resides in the insect midgut together with infecting malarial parasites, can be cultured and labelled with GFP for following the dynamics of infection, multipies 200-fold following ingestion and, most importantly, can be transformed to secrete anti-*Plasmodium* effector molecules [121].

The classic paper of Wang et al. [121] describes the use of the *E. coli* HlyA secretion system to separately transform *P. agglomeratus* strains with eight anti-*Plasmodium* effector molecules. The expression and secretion of each of the effector proteins by the recombinant *P. agglomeratus* were confirmed by Western blotting. To test the effects of the transformed bacteria on infection of *An. gambiae* and *An. stephensi* by *Plasmodium falciparum* or *P. berghei*, the bacteria were fed to the mosquitoes on cotton pads soaked with bacteria suspended in 5% sucrose. After 32 h, the mosquitoes were given an infected blood meal, and 8 days later numbers of oocysts formed were counted. Five of the effector proteins secreted by the transformed *P. agglomeratus* significantly inhibited parasite development by up to 98% for scorpine or (EPIP)_4_ (*Plasmodium* enolase–plasminogen interaction peptide). Combinations of two types of effectors were no more effective at parasite inhibition than individual proteins; this, however, does not consider possible enhanced resistance of *P. agglomeratus* protein combinations to parasite mutation and evolution. The importance of this study is that the engineered *P. agglomeratus* were equally effective at inhibiting malarial parasites in both *An. gambiae* and *An. stephensi* so that any reproductive or behavioural barriers that may exist between isolated vector populations in the wild will not affect paratransgenesis [121]. Progress in the widescale use of *P. agglomerans* for mosquito control has not advanced rapidly since the Wang et al. paper [121], probably as a result of the unresolved problem of driving the bacteria into wild mosquitoes [120] (see section Concluding remarks including safety and environmental concerns of present article) and the continued resistance to the release of engineered microorganisms into the environment. In addition, reports of *P. agglomerans* causing secondary human infections in bones and joints as well as pathogenic strains in some crops have to be considered [204, 205], although it is unlikely that these pathogenic bacteria are the same as those isolated from insects.

##### *Serratia*

*Serratia* spp. have been widely reported in the midguts and tissues of *Anopheles*, *Aedes* and *Culex* mosquitoes as well as in many non-vector insect orders [206, 207]. Interest in *Serratia* has previously been centred around the potential use of these bacteria for controlling the malarial parasite in the mosquito host (e.g. [208]). This *Plasmodium*-inhibitory activity of *Serratia* spp. has been shown to result from multiple mechanisms, including the upregulation of the mosquito immune system [209] by the direct production of anti-malaria factors by the bacteria themselves [157, 209–211], and by blocking ookinete penetration through the vector midgut epithelial cells [210]. The possibility therefore exists of transfecting mosquitoes with specific strains of *Serratia* to control malaria although much additional work is required and *Wolbachia*-based strategies have been given priority at the present time [62–64]. However, as mentioned earlier in this article, the use of *Wolbachia* in paratransgenesis has not been developed so far as the bacterium cannot be genetically transformed and is difficult to culture (it is an obligate intracellular symbiont) [56, 57, 212]. Reveillaud et al. [213], however, reported *Wolbachia* from four wild *Culex pipiens* mosquitoes carrying a plasmid (pWCP), indicating that future paratransgenesis utilising *Wolbachia* may be possible.

The potential use of *Serratia* for paratransgenesis has also been recognised [121, 122, 211, 214, 215]. Wang et al. [122] previously engineered natural symbiotic *Pantoea agglomerans* to secrete anti-*Plasmodium* effector molecules (see section *Pantoea agglomerans* (= *Enterobacter agglomerans* of present article) but failed to address the problem of infecting wild mosquito populations. These researchers then discovered, in *Anopheles stephensi*, a strain of *Serratia* called AS1 which has no fitness costs following engineering to produce anti-*Plasmodium* effectors in *An. stephensi* or *An. gambiae* [122]. Using fluorescent markers incorporated into the bacteria, the colonisation of the mosquitoes by *Serratia* AS1 was studied in laboratory cage experiments. In just one mosquito generation, AS1 was venerally transmitted horizontally from males to females during mating and then vertically to the offspring. It also survived larval metamorphosis to multiply in the mosquito midguts and other organs for multiple generations. The transformed *Serratia* AS1, producing multiple anti-*Plasmodium* effectors, were also fed to mosquitoes and inhibited the *Plasmodium falciparum* life-cycle [121]. Koosha et al. [215] also used *Serratia* AS1 labelled with mCherry fluorescent protein to study the acquisition of bacteria by arthropod vectors, including *An. stephensi*, *Culex pipiens*, *C. quinquefaciatus* and *C. theileri*. Subsequently, all adult mosquitoes took up the bacteria from the host skin during blood-feeding and from the water when larvae. The larvae then transferred them to the adults transstadially and these finally returned them back to the water during egg-laying (Fig. 2).

More recently, Huang et al. [134, 162] have addressed possible regulatory concerns about the release of engineered bacteria into the environment and any uncertain consequences that might occur. They have designed a self-limiting paratransgenesis using *Serratia marcescens* AS1 and *An. stephensi*. In this system, plasmids were used to transform *Serratia* AS1 bacteria, but these plasmids were lost in 130 generations so that the bacteria returned to wild type. Thereby, the plasmids were lost in three mosquito generations. Equally important, for satisfying regulators, there was no evidence, following feeding of plasmid-transformed AS1 to vector insects or their incubation in culture with high concentrations (10^12^) of *E. coli* or *P. agglomerans*, for horizontal transfer of plasmid genetic material to other bacteria (Table 1) [134, 162].

##### *Enterobacter*,* Escherichia*,* Chromobacterium*,* Elizabethkingia* and* Pseudomonas*

Apart from *Asaia*, *Pantoea* and *Serratia* described above, *Enterobacter*,* Escherichia*,* Chromobacterium*,* Elizabethkingia* and* Pseudomonas* are examples of other members of the phylum Proteobacteria with potential use in paratransgenesis but for which less published information is available. *Enterobacter* infections in *Anopheles arabiensis* and *An. gambiae*, without engineering, were shown to block *Plasmodium falciparum* parasites [216, 217]. Also, following an infected bloodmeal, *Enterobacter cloacae* rapidly colonised the midgut of *An. stephensi* and the bacteria were amenable to transformation but only weakly transferred from larvae to adults so of no use for multigeneration recycling [218]. *Escherichia coli* was transformed and shown to inhibit *Plasmodium berghei* in *An. stephensi* but the effect was suboptimal, the effector molecules stuck to the bacterial surface and the *E. coli* strain used survived poorly in the mosquito gut [81]. *Chromobacterium* isolated from the midgut of *Aedes* and *Anopheles* mosquitoes has been shown to have both anti-*Plasmodium* and anti-dengue virus activity in vitro and to kill *Anopheles coluzzii* after infective feeding [153, 219, 220]. These toxic properties are probably at least partially due to a secreted protease, suggesting that the bacteria could be engineered to produce this effector in the midgut of mosquitoes [220].

*Elizabethkingia* is also common in anopheline mosquito microbiomes from western Thailand [179], can be transmitted transstadially and has been transformed to re-infect *Anopheles* mosquitoes [221]. It is, however, a potential human pathogen with resistance to some antibiotics so caution would be required [222]. Similar pathogenic concerns exist for *Pseudomonas* isolated from the common Asian vector, *Anopheles culicifacies* [160] and from *Culiseta longiareolata* [223]. It is present in both larvae and adults and so may be transstadial and, depending upon the species, may be a possible candidate for paratransgenesis.

#### Mosquito microbiomes: viruses and fungi

The choice of symbiotic microorganisms for developing paratransgenesis in mosquitoes has also now been extended from bacteria to include viruses and fungi, although the majority of studies have utilised bacterial symbionts (e.g. [83, 86]). Details of potential viral and fungal candidates for paratransgenesis have been discussed in previous sections (see section Analysis of microbiomes of present article). Gurung et al. [224] believe that focussing attention too much on bacteria in the microbiome and ignoring the other microbial components, such as the fungi, viruses, archaea and protozoans, may hamper full understanding of the true impact of the microbiome on the insect pest. This is just as likely to apply to the effect of the microbiome on invading parasites.

#### Mosquito microbiomes: RNAi-based paratransgenesis

This is a relatively new technique in vector insects in which the transformed symbionts deliver dsRNA instead of the usual effector proteins to silence or knock down a specific host or even parasite genes (for more details, see section Transformation of symbionts of present article). In addition, the use of RNAi for the control of mosquitoes and malarial parasites is growing [115] although technical difficulties exist. For example, both *Aedes aegypti* and *Ae. albopictus* contain 10 dsRNases which would rapidly degrade any dsRNA in the gut lumen [225]. Subsequent dsRNA knockdown of two key dsRNases resulted in a high efficiency of gene knockdown by dsRNA targeting a cyan fluorescent protein (CFP) reporter gene given by feeding [225]. Another way to enhance the survival of the dsRNA in the insect would be to use symbiotic bacteria to both protect and produce the dsRNA rather by feeding or injecting naked dsRNA [74, 114, 226].

For a summary of this section, see Table 2.

### Triatomine microbiomes

There are approximately 152 described species of triatomine bugs, of which 67 occur in Brazil [227]. About half of these species can carry *Trypanosoma cruzi*, the causative agent of Chagas disease. This disease also induces chronic inflammation of the heart, colon and nervous system, and the parasite DNA can undergo vertical transmission to the progeny of mammals [228].

There has been an increased interest in the microbiome of these insects since details of the roles of the component bacteriome in the host physiology and interactions with the flagellate parasite, *T. cruzi*, were revealed (e.g. [54, 229–236]). Resistance to conventional insecticides also stimulated research on the triatomine microbiome [237], resulting in the introduction of paratransgenesisis as an alternative control technique, for the first time in vector insects, in *Rhodnius prolixus* (e.g. [69]).

The expansion in this research area with triatomines was also mediated by the application of molecular techniques, including high-throughput* 16S* rRNA and, more recently, next-generation sequencing and bioinformatics, to identify most members of the microbiome (e.g. [233, 234, 238–252]). These studies have looked at the microbiomes of varying numbers of wild and laboratory-reared triatomine species with and without parasites. In addition, these insects were from different geographical regions and ecological niches, at various developmental stages, and involved different feeding regimes, sexes, physiological states and tissues, utilising alternative sampling and analytical techniques. Therefore, and similar to the situation in mosquitoes (see section Mosquito microbiomes in present article), generalisations have been difficult to make. Fortunately, Duarte Silva et al. [253] and Salcedo-Porras et al. [234] have recently analysed and rationalised the results of some of these studies in detail although many contradictions still exist. In addition, Brown et al. [250] designed their research to eliminate some of these variables by, for example, using five wild *Triatoma* species sampled from the nests of white-throated woodrats in which all five instars plus adults could be found occasionally, as well as other species, all feeding on the same blood source. Some basic but not universal conclusions that can be drawn from these papers on the triatomine microbiome are as follows:

i. Most triatomines have a low diversity of bacterial genera in comparison with other insects, but variability exists between species even when they originate from identical environments, such as the same nest [245].
ii. The triatomine microbiome, which shows similarities to other vector insects [246], contains members of the Gram-negative phylum Proteobacteria (e.g. *Serratia*, *Enterobacter*, *Pantoea*, *Acinetobacter*, *Arsenophonus*, *Pseudomonas* and *Wolbachia*) and the Gram-positive phylum Actinobacteria (including *Rhodococcus*, *Nocardia*, *Dietzia*, *Gordonia*, *Corynebacterium and Mycobacterium*), which together make up 20–50% of the microbiome. In addition, Gram-positive Firmicutes (20%; e.g., *Enterococcus*, *Staphylococcus*, *Bacillus*) and Gram-negative Bacteroidetes (e.g. *Proteiniphilum*; 5–7%) are also present [234]. However, in only three species of triatomines were mutualistic symbionts identified, all Actinobacteria [254].
iii. Many Proteobacteria, but particularly the Enterobacteriales (e.g. *Arsenophorus*, *Serratia* and *Enterobacter*) and Corynebacteriales (e.g. *Rhodococcus*, *Nocardia*, *Dietzia*, *Gordonia*, *Corynebacterium and Mycobacterium*), are present in multiple triatomines.
iv. Similar changes occur in the microbiome in wild triatomines throughout development and from one gut compartment to another. These involve a change from high microbiome diversity to low diversity from first instars to adults which are often dominated by a single bacterial genus, including *Dietzia*, *Mycobacterium* or *Proteiniphilum* [245].
v. Wild insects naturally infected with *T. cruzi* have a more diverse microbiome than uninfected wild insects or infected or uninfected cultured insects [164, 241], but see [244, 247].
vi. *Rhodnius* spp. and *Triatoma infestans* are the only triatomines in which *Wolbachia* has been reported in both wild and laboratory populations [234, 249, 252].

Some of the above bacteria, and many more reported in the papers cited previously, would be good candidates for paratransgenesis, assuming that they can be cultured, are non-pathogenic for humans or animals and can be genetically manipulated with no adverse effects on their stability or fitness or on the host vector. *Serratia*, *Pantoea* and *Enterobacter* have already been tested in mosquitoes (see section Mosquito microbiome: native endosymbiotic bacteria in paratransgenesis of present article) as have *Corynebacterium*, *Escherichia and Rhodococcus* in triatomines (see section Transformation of symbionts of present article). Another factor in choosing bacteria for paratransgenesis is to select a species with high GC-content since, in the triatomine gut, bacterial species with high GC-contents have been shown to outcompete those with low GC-content [242].

### Triatomine microbiomes—native endosymbiotic bacteria in paratransgenesis

The pioneering steps in the development of paratransgenesis were made with the triatomine, *Rhodnius prolixus*, utilising the genetically transformed actinimycete bacterium, *Rhodococcus rhodnii*, to deliver the trypanolytic antimicrobial peptide (AMP), cecropin A [66–71, 77]. The target of this peptide was *Trypanosoma cruzi* (for details see section Development of paratransgenesis of present article). The use of cecropin A was a successful proof of concept study and led to further experiments with other effector molecules in order to improve both the efficiency of parasite killing and reduce the likelihood of resistance developing (Tables 1 and 3). Several other single AMPs tested in vitro killed *T. cruzi*, but when AMPs were combined, for example, apidaecin with cecropin, melittin or magainin, the results with all pair-wise combinations achieved 100% lethal concentration (LC_100_) levels, in contrast to the single AMPs [80]. Strains of *R. rhodnii* have been transformed in vitro to produce these AMPs although the results of in vivo experiments with *T. cruzi* have not appeared. Instead, single-chained antibodies and recombinant β-glucanase have been developed as effector molecules against *T. cruzi* [70, 72, 255, 256] (Table 3).

Durvasula et al. [72, 255] and Hurwitz et al. [82] have proven the feasibility in *R. prolixus* and *Triatoma infestans* of the expression and secretion by engineered symbionts of functional fragments of the murine three-domain antibody (rDB3) capable of recognising and binding to progesterone. For this, the genetically engineered symbionts *R. rhodnii* and *Corynebacterium* sp., respectively, for *R. prolixus* and *T. infestans*, expressed and secreted functional fragments of rDB3 into the insect gut. The recombinant strains of *R. rhodnii* and *Corynebacterium* sp*.* were added to the blood meal of aposymbiotic first instar nymphs and shown to synthesise and secrete rDB3 for 6 months of the study [72, 82, 255]. Subsequently, small antibody molecules were produced against the sialyl-Tn and sialyl-(le)a surface glycans of *T. cruzi* [257] and shown by confocal microscopy to specifically bind to fixed *T. cruzi* epimastigotes [76].

Regarding recombinant β-glucanase as an effector molecule in paratransgenesis, the surface of *T. cruzi* is covered in a layer of mucin-like glycoproteins that are probably essential for the in vivo development of the parasite by mediating its binding to the triatomine midgut and hindgut cells [258, 259]. Jose et al. [256] concluded that disruption of the surface glycocalyx of *T. cruzi* would therefore inhibit the development of the parasite. To prove this, *R. rhodnii* was transformed to express β-1,3-glucanase, as this protease was previously shown to efficiently promote cells lysis in *T. cruzi* [76, 256]. Jose et al. [256] inserted the complementary DNA (cDNA) encoding the *Oerskovia xanthineolytica* β-1,3-glucanase gene (i.e. *Arthrobacter luteu* strain 73–14) into plasmid pRrExpA used for manipulation of *R. rhodnii*. In vitro assays performed with *T. cruzi* incubated in culture medium together with the recombinant *R. rhodnii* showed a more than 80% inhibition of parasite growth. The results proved the efficiency of the recombinant bacteria-expressed β-1,3-glucanase in lysing *T. cruzi* cells. Therefore, recombinant β-1,3-glucanase represents a valuable additional effector molecule for paratransgenesis against *T. cruzi* in its triatomine hosts.

These studies show that it is potentially possible to produce effector molecules targeting a range of different sites in *T. cruzi* to reduce the likelihood of the parasite becoming resistant.

A significant problem for paratransgenetic control of diseases in insect vectors is the delivery of transformed symbionts to specific wild insect vector populations in the field [127]. In the case of the triatomine, *R. prolixus*, this problem is readily solved with coprophagy spreading the transformed symbionts naturally to the whole population. The newly emerging *R. prolixus* nymphs are aposymbiotic (devoid of gut symbionts) but soon become infected from the surrounding faeces produced by the whole colony. To study transgenesis in simulated field conditions, Durvasula et al. [73] used large cages containing local Guatemala dirt and thatch with panels impregnated with CRUZIGUARD, a paste containing transformed *R. rhodnii* suspended in sterile phosphate-buffered saline plus guar gum powder. Newly emerging first instar *R. prolixus* from eggs of field-caught insects were housed in the cages, and guts were sampled at the third and fifth instar and adult stages and tested for transformed *R. rhodnii*. Approximately 56% of the experimental insects contained the transformed bacteria to the exclusion of other competing bacteria in the environment [73]. In addition, when nymphs were allowed to develop for 9 months in the cages, approximately 50% of adults were shown to contain transformed *R. rhodnii*. This technique could potentially be used along with insecticides to prevent reinfestations of homes.

#### Triatomine microbiome: RNAi-based paratransgenesis

It is significant that it has been nearly 30 years and > 20 years, respectively, since the pioneering works of Beard et al. [66] and Durvasula et al. [71] were published on paratransgenesis in *R. prolixus* and, although significant advances have been made, approval for use in the field has yet to be obtained.

Recent work on the use of symbiotic bacteria to deliver dsRNA for knockdown of specific genes in triatomines represents a significant step forward (see section Transformation of symbionts in present article) [74, 111, 113, 260]. This technique has also been adapted for development in mosquitoes and may help to satisfy the regulatory process for the release of transgenic bacteria in the field (see section Concluding remarks including safety and environmental concerns of present article).

For a summary of the above, see Table 3.

**Table 3:** Genetic manipulation of bacteria with potential use for paratransgenic control of *Trypanosoma* and *Leishmania* spp.

Transformed microbes used + effector genes	Insect vectors	Experimental results with transformed microbes	Key references
*R. rhodnii* + pRr1.1 shuttle plasmid with antibiotic resistance	*R. prolixus*	First proof of concept in insect vectors. Successful transformation and maintenance of symbiotic bacteria in vector	[66, 67, 70]
*R. rhodnii* + RrThioCec-(*transformed R. rhodnii* + cecropin A)	*R. prolixus*	Elimination or reduction of *T. cruzi *in vivo	[69, 71]
*R. rhodnii* + rDB3 antibody fragment	*R. prolixus*	Secretion of antibody fragments into gut lumen in vivo	[72]
*Corynebacterium* sp. + rDB3	*Triatoma infestans*	Secretion of antibody fragments into gut lumen in vivo	[82, 255]
*R. rhodnii* + recombinant *Arthrobacter luteus* β-1,3-glucanase	Potential additional effector in *R. prolixus *in vivo	Lysates of β-1,3-glucanase transformed *R. rhodnii* kill *T. cruzi *in vitro	[256]
*R. rhodnii* + rDB3 antibody fragment	*R. prolixus*	Semi-field simulation of transgenic bacteria spread in Cruzigard	[73]
*E. coli* + dsRHBP + dsCAT	*R. prolixus*	Proof of concept with transgenic symbiont -mediating RNAi in adults and nymphs	[111]
*R. rhodnii* + dsNP1 *R. rhodnii* + dsNP2 *R. rhodnii* + dsVg	*R. prolixus*	Proof of concept with transgenic symbiont -mediating RNAi in aposymbiotic nymphs	[74, 113, 260]
*Sodalis glossinidius*^-fp^	*Glossina morsitans morsitans*	Transfection study to progeny	[287, 288]
*S. glossinidius*^-gfp^	*G. m. morsitans* *Glossina fuscipes fuscipes*	Reciprocal transinfection occurs with no fitness costs	[270]
*S. glossinidius* + Nb_An46	*G. m. morsitans*	The nanobody was expressed in vivo by the transformed *Sodalis*	[289, 290]
*S. glossinidius*^-gfp^	*G. m. morsitans*	Much impoved bacterial colonisation of progeny	[291]
*S. glossinidius*	*G. m. morsitans*	Paratransgenesis combination advocated with sterile insect technique	[294]
*Bacillus subtilis*^-gfp^	*Phlebotomus argentipes*	Laboratory transfection study in larvae and transstadial transmission	[330, 331]
*Enterobacter cloacae*^-DR^	*Phlebotomus papatasi*	Laboratory transfection study with limited tranasstadial transmission	[325]

*gfp green fluorescent protein, DR Enterobacter cloacae * expressing red fluorescent protein plus defensin (EC-DR),* dsRHBP + dsCA * dsRNA for*Rhodnius* heme-binding protein (RHBP) and catalase (CAT),* dsNP1* dsRNA for Nitrophorin-1,* dsNP2* dsRNA for Nitrophorin-2,* dsVg * dsRNA for Vitellogenin,* Nb_An46 * a potent trypanolytic nanobody, i.e. Nb_An46. (Nanobody®)

### Tsetse fly microbiomes

Tsetse flies (genus *Glossina*) are viviparous with 30–33 species and subspecies having been described [58]. These are usually divided into the *Morsitans*, *Palpalis* and *Fusca* groups containing various species and subspecies which are particularly important medically and economically due to transmission of African trypanosomes [58]. African trypanosomiasis affects both people and their livestock (e.g. [261, 262]). Cases of human sleeping sickness rapidly declined from 1997 to 2019, with many countries reporting no new cases for the last decade [14]; however, there is a constant risk of re-emegence from animal and human reservoirs. There are also no vaccines for sleeping sickness, and chemotherapy is both expensive and toxic, and the parasites are showing increasing resistance [262, 263].

A number of studies have been made of the tsetse microbiome [264–279]. The results indicate that tsetse flies host a large range of bacterial species, often including four maternally transmitted endosymbiotic bacteria present in both wild and laboratory-reared flies (e.g. [269, 274]), namely *Wiggleworthia glossinidia*, *Sodalis glossinidius*, *Wolbachia* and *Spiroplasma. Wiggleworthia glossinidia* occurs intracellularly in bacteriocytes in the anterior gut to produce supplements for tsetse nutrition and often dominates to form 34.5–99.8% of the microbiome [272]. *Sodalis glossinidius* is present in the midgut, muscle, fat body, salivary glands and milk glands, while *Wolbachia* is found in the ovaries, with both varying greatly in incidence (e.g. [264, 269, 274, 280]). *Spiroplasma* is a more recently discovered transovarially transmitted endosymbiont present in the *Palpalis* group and culturable in vitro [281–283]. In addition, the tsetse microbiome has a diversity of commensal bacteria from the environment, which usually account for < 1% of the bacteriome [269, 273]. The number and incidence of these bacterial species vary from one study to another depending both on the methodology used and the species of tsetse fly sampled (e.g. [272]). For example, of the 103 species of bacteria described in *Glossina palpalis palpalis* by Jacob et al. [272], Gram-negative bacteria predominated as did the phylum Proteobacteria (97% of isolates), with members of the phyla Bacteroidetes, Actinobacteria and Firmicutes also represented and the microbiome showing some resemblance to those of *Anopheles* and *Aedes.* In contrast, with the *Glossina pallidipes* microbiome, Malele et al. [269] reported that of 113 isolates, the descending order of prevalence was Firmicutes (86.6%), Actinobacteria (7.6%), Proteobacteria (5.5%) and Bacteroidetes (0.3%). Examples of bacteria identified in *Glossina* microbiomes include the genera *Bacillus*, *Serratia*, *Pantoea*, *Acinetobacter*, *Arthrobacter*, *Enterobacter*, *Enterococcus*, *Providencia*, *Sphingobacterium*, *Chryseobacterim*, *Exiguobacterium*, *Lactococcus*, *Staphylococcus*, *Pseudomonas*, *Spiroplasm* and *Xylella* (e.g. [268, 275, 278, 283, 284]).

#### Tsetse fly microbiomes: native endosymbiotic bacteria in paratransgenesis

The four maternally transmitted endosymbiotic bacteria, *Wiggleworthia*, *Sodalis*, *Wolbachia* and *Spiroplasma*, are the dominant symbionts in *Glossina* and are transmitted maternally so that these were the natural candidates for developing paratransgenesis in tsetse flies. Of these symbionts, however, *Wigglesworthia* cannot be cultured, *Wolbachia* is not genetically transformable or easily cultured and *Spiroplasma* is a recent discovery. Thus, to date, *Sodalis*, which can be both cultured and transformed, has been utilised for paratransgenesis-related experiments in *Glossina* [13, 261, 264, 270, 285–295]. There is also evidence that some *Sodalis* genotypes can favour the establishment of trypanosme infections in tsetse flies by inhibiting the trypanocidal activity of the *Glossina* midgut lectin (e.g. [269, 274, 296–298]), although this varies with species, location and study [271, 276].

As a first step in developing paratransgenesis in *Glossina*, Cheng and Aksoy [287] studied the transmission of S-symbionts (presumably *Sodalis*) to the vector progeny. For this, they injected transformed S-symbionts expressing GFP into the hemolymph via the thorax of mated female *G. m. morsitans* and collected the F1 and F2 progeny. The gut tissues of the progeny were sampled and analysed for S-symbionts by PCR amplification using GFP-specific primers. The progeny haemolymph was also cultured and tested for the presence of GFP-expressing symbionts. Both techniques detected the transformed S-symbionts in the F1 and F2 flies to confirm the vertical transmission from mother flies. The presence in the milk glands of fluorescent recombinant symbionts also indicated that the route of transmission was from the haemolymph to the intrauterine larvae via secretion of these glands [287]. This work indicated two important factors necessary for successful paratransgenesis in tsetse flies: (i) the vertical transmission of the symbiont from the mother to the progeny and (ii) the ability of the transformed symbiont to express the heterologous gene effectively and stably in the insect vector [287].

An additional study advancing paratransgenesis in *Glossina* is that by Aksoy et al. [288] who describe why *Sodalis* is well-suited for paratransgenesis in tsetse flies since it occurs in the gut together with the trypanosomes, can be cultured, is resistant to trypanocidal peptides and can be genetically transformed and transmitted to the progeny.

Transformation in *Sodalis* has previously been performed with plasmids [286, 289–291]; however, this may not be optimal for field experiments as plasmid maintenance may require constant selection. This possibility has been recognised by both Aksoy et al. [288] and De Vooght et al. [291] who have utilised alternative methods. Aksoy et al. [288] undertook transformation using a piece of non-replicating circular DNA, with a sequence homologous to the desired chromosomal loci, which allowed transgenic symbionts to be maintained without selection. De Vooght et al. [291] also used the chromosomal expression of a reporter gene under the control of a native or a heterologous constitutive promoter. More recently, it was also discovered in *Sodalis glossinidius* that conjugation can be used as a DNA delivery method to conduct forward and reverse genetic experiments [299].

In another fundamental study providing key background information for paratransgenesis in tsetse flies, reciprocal swopping of *Sodalis* populations between *G. fuscipes fuscipes* and *G. morsitans morsitans* flies had no dentrimental fitness effects compared to the wild-type flies in terms of fecundity and longevity [270]. For these experiments, newly emerged adult flies were fed with blood plus antibiotic to clear the bacteria and then injected with the *Sodalis* strain from the other *Glossina* species of the pair, i.e. reciprocal transinfection. In these flies, the bacteria were also successfully transmitted to their progeny. These results indicate that in the field it would be possible to simultaneously control African trypanosomatid transmission by different *Glossina* species with a single recombinant strain of *Sodalis* expressing anti-parasitic effectors [270].

The next step in paratransgenesis is to identify effector molecules produced by the transformant symbionts and capable of expression and killing trypanosomes in *Glossina* without loss of fitness of the symbiont or the host insect. *Glossina* attacin is one possibility as it can kill trypanosomes both in vitro and in vivo without affecting *Sodalis* [300]. The first report, however, of the successful use of an anti-trypanosome effector molecule expressed in vitro and in vivo in *Glossina* was of a single domain antibody (Nanobody® molecules), Nb_An33, targeting conserved epitopes of the variant surface glycoprotein (VSG) of *Trypanosoma brucei* [290–292, 301].

For this work, a FliCpelBNb46fliC plasmid-based *Sodalis* strain was initially produced that expressed the trypanolytic nanobodies. This system was shown to be highly stable in vitro after 27 generations; therefore, the ability of the recombinant *Sodalis* (rec*Sodalis*) to colonise the *G. morsitans morsitans* tissues after intrathoracic injection was assessed. In order for the rec*Sodalis* to succesfully colonise the flies, it was first necessary to remove the wild-type *Sodalis* present in the recipient *Glossina* with streptomycin [290]. The rec*Sodalis* also persisted at high densities in the thorax and gut tissues for up to 28 days without affecting the population of *Wigglesworthi*a, an essential *Glossina* endosymbiont, or the fecundity of the flies. Furthermore, the recombinants were also transmitted to the F1 progeny, but at only very low levels. Finally, nanobody concentrations were quantified over time with a VSG-binding enzyme-linked immunosorbent assay; functional Nb_An33 was found to accumulate in the haemolymph and thorax, indicating the expression of the injected transgene [290]. It was calculated that the levels of nanobody produced would probably be sufficient to deal with the average parasitiaemia of 10^3^ *T. brucei* in cattle.

The above results are very encouraging for the development of paratransgenesis in tsetse flies although the transmission levels to the F1 progeny were very low. This could be due, as mentioned above, to instability of the plasmid and the need for a more stable transformation system. De Vooght et al. [291], therefore used chromosomally GFP-tagged rec*Sodalis* to colonise various tissues of tsetse flies and follow their transmission to the F1 progeny using different infection procedures. Injecting adults intrathoracically resulted in high-density colonisation of the tissues but limited infection of the reproductive organs (milk glands, etc.) and no vertical transmission to the progeny. Oral feeding of *Glossina* with rec*Sodalis* also failed to infect either the adults or the offspring. Finally, injection of the third instars gave stably infected adults and subsequent vertical colonisation of the next generations of flies. Apparently, in the larvae, certain invasion and motility genes are upregulated, such as *invC* and *fliC* and *motA*, and these may be required for vertical transmission (e.g. [302]).

These studies are important steps forward in the development of paratransgenesis in tsetse flies. In the future, populations of the resistant rec*Sodalis*-infected tsetse flies might be driven into suscepible field populations utilising the cytoplasmic incompatibility induced in flies by *Wolbachia* infections [288]. This possibilty has been modelled by Gilbert et al. [293] with human African trypanosomes that could potentially be eliminated over a 25-year period if colonisation by *Wolbachia* had minimal fecundity or mortality impacts on tsetse flies. The chance of recombinant *Sodalis* vertical transmission was also > 99.9%. In addition, control of African trypanosomiasis coud be mediated by paratransgenesis in tsetse flies combined with the the sterile insect technique, as advocated by Demirbas-Uzel et al. [294].

### Sand fly microbiomes

About 500 sand fly (phlebotomine) species are known, of which more than 90 transmit leishmaniasis. The main vectors of human leishmaniasis are species and subspecies of *Phlebotomus* in the Old World and *Lutzomyia* in the New World [12]. East Africa, Brazil and India are particularly affected by visceral (fatal) leishmaniasis. Sand flies also vector several pathogenic viruses, including phleboviruses causing encephalitis, meningitis and haemorrhagic fever [303, 304]. Like mosquitoes, the female sand fly needs blood for egg development and transmits the pathogens during feeding. There are > 20 species of *Leishmania*, with most infected people showing few symptoms. In 2019, 97 countries were endemic with > 1 billion people at risk of infection and almost 1 million new cases of cutaneous leishmaniasis occurring annually [12]. An inactivated/killed *Leishmania major* vaccine with Bacillus Calmette–Guérin was developed but failed to protect against the disease [305]. Recently, scientists have characterised a new strain of *Leishmania* for use in a human infection model and are seeking volunteers for an initial trial [306]. Medicines for treatment of leishmaniasis may be limited in poorer countries, with toxicity and emerging resistance problematic and complications arising from HIV co-infections [12]. In addition, pesticide resistance by sand flies has also been detected [307]. The conclusion, therefore, is that new tools are also required to control this disease [308].

The main requirements for the development of paratransgenesis in sand flies are the same as those in other vector insects (summarised in section Requirements for successful paratransgenesis of present article)**,** and have been reviewed for phelbotamines by Wijerathna et al. [309]. The prime requirement is the identification of appropriate bacterial species in which to develop the technique. There have been many studies on the commensal bacteria in *Lutzomyia* (e.g. [310–317]), and *Phlebotamus* (e.g. [97, 318–328]). Fortunately, the extensive reviews of sand flies by Telleria et al. [315], Wijerathna et al. [309] and Omondi and Demir [329] provide summaries of many of these studies which, taken together, identify numerous species of bacteria.

These bacteria are dominated by Gram-negative members belonging to the phylum Proteobacteria, with the Gram-positive phyla Firmicutes and Actinobacteria also represented, and include *Ochrobactrum*, *Serratia marcescens*, *Klebsiella*, *Enterobacter*, *Escherichia coli*, *Pseudomonas aeruginosa*, *Pantoea agglomerans*, *Acinetobacter baumannii*, *Methylobacterium*, *Wolbachia*, *Spiroplasma*, *Enterococcus faecalis*, *Staphylococcus aureus*, *Bacillus cereus*, *B. anthracis*, *B. subtilis* and *B. megaterium* [309, 315, 319, 328]. Several of these species belong to the phylum Proteobacteria, with *Serratia *and* Enterobacter* in the family Enterobacteriales and *Pseudomonas* forming core taxa, as has been described above in some other vector insects. There is also divergence in the bacteriome between wild and laboratory-reared sand flies as well as a reduction in taxa from *Leishmania*-parasitised insects [314].

#### Sand fly microbiome: native commensal bacteria in paratransgenesis

Regarding the choice of bacteria for paratransgenesis in sand flies, *S. marcescens*, *P. agglomeratus* and *Enterobacter cloacae* have already been tested in mosquitoes while *E. coli* has been utilised in triatomines (see preceding sections on different vectors). These isolates have been reported in sand flies so that the appropriate technology could be tranferred for use. There are, however, reports of pathogenicity for strains of these bacteria in humans so that alternative species have been identified for developing paratransgenesis in sand flies. In fact, the first experiment on the transstadial passage of commensal bacteria in sand flies was successful but with a potentially major pathogen, *Ochrobactrum* sp. in *Phlebotomus duboscqi* [319]. Subsequently, Hillesland et al. [320] identified several particularly suitable non-pathogenic bacteria, including *B. subtilis*, *B. megaterium* and *Brevibacterium linens*, for developing paratransgenesis in *Phlebotomus argentipes*. All three species are sold as probiotics, with *B. megaterium* also having potential as a biofertiliser for spreading in the environment [320].

The first study of the possibility of genetically manipulating sand fly commensal bacteria capable of effectively colonising the insect and remaining permanent throughout the life-cycle was conducted by Hurtwitz et al. [330, 331] with *B. subtilis*, previously isolated from *P. argentipes.* For this work, *B. subtilis* expressing a GFP reporter gene was added in the diet and offered to fourth stage sand fly larvae; the insects were then dissected and the midgut homogenates analysed for colonisation by recombinant bacteria using PCR and colony-forming units. The *Bacillus*^-gfp^ colonised the fourth stage larvae of *P. argentipes* effectively and stably and could be recovered throughout the different stages of insect development. The recombinant strain was isolated from all larvae and pupae and from 75% of adults. In addition, sand fly adult emergence over 18 days was similar in *B. subtilis*-treated larvae and controls [330]. There was also no apparent horizontal transfer of the plasmid used for transformation to other bacteria in the gut. Whether the bacteria affected the female sand fly fecundity or were transmitted to the progeny was not determined. The transformation of *B. megaterium* to express a single chain antibody has also been reported [320], as has the development of melittin and human histone 2B as anti-*Leishmania* effector molecules [332].

More recently, Abassi et al. [325] also studied the transformation of the commensal sand fly bacterium, *Enterobacter cloacae* subsp. *dissolvens*, to express a defensin and colonise *Phlebotomos papatasi*. This defensin is of plant origin and able to kill parasites but not bacteria [325]. The bacteria were transformed with a red fluorescent protein plus defensin plasmid. When first instar larvae were fed just once on a diet containing the transformed bacteria, the latter could be detected up to 36 days post-feeding but there was no transstadial transmission to adult sand flies. This may be due to the loss of the bacteria during pupation and/or the inability of bacteria to colonise the gut due to physiological changes, such as those of the pH gradients [333].

In conclusion, much additional work is required with sand flies before paratransgenesis can be fully instigated for field trials.

## Concluding remarks including safety and environmental concerns

This overview describes progress in the development of paratransgenesis in vector insects and shows that the majority of the research, not surprisingly, is currently focussed on mosquitoes. As mentioned previously, the original technique was pioneered nearly 30 years ago and although significant technical advances have been made with regulatory laws in mind, approval for use in the field has yet to be obtained. Many of these advances are shown in Table 1, but reasons for failure to have paratransgenesis adopted into IPM programmes are manifold. Some of the problems to be addressed have been identified previously (e.g. [34, 69, 83, 334, 335]) and their partial resolutions are indicated in Table 1.

To gain support from regulatory bodies, there are important requisites, including proof that the released transformed bacteria are stable and non-pathogenic to other animals and humans, and that they do not infect harmless insects by horizontal gene transfer. In addition, it is necessary to have detailed studies of the tripartite interactions of vector microbiomes from different ecosystems with vector immunity and invading parasites in order to identify suitable candidate microbes for paratransgenesis [83, 157]. Furthermore, ideal properties of transgenic bacteria include ease of spread into wild populations, the ability to pass transstadially and the ability to persist in different generations of the vector without inducing resistance (e.g. [336, 337]).

Table 1 gives examples of studies satisfying many of these requirements. For example, horizontal gene transfer in vivo was shown by Huang et al. [134] not to occur in *Anopheles stephensi* infected with fluorescent *Serratia.* Additionally, Matthews et al. [133] modelled the chances of horizontal gene transfer in the gut of *Rhodnius prolixus* and predicted the frequency of this process occurring at less than 1.14 × 10–16 per 100,000 generations with a 99% certainty level. Other attempts to allay fears about the effects of transgenic bacteria released into the environment and prevent their excessive spread have also been reported using microencapsulation techniques (Table 1). For example, in semi-field trials with *Rhodococcus rhodnii* in *R. prolixus*, the bacteria were enclosed in guar gum to form CRUZIGARD [71] while with *Pantoea agglomerans* in *Homalodisca vitripennis* an alginate hydrogel was used [128]. The work of Huang et al. [134] on *Serratia* ASI in *An. stephensi* also specifically addresses the concerns of regulators about the effects of the release of transgenic bacteria on the environment if something goes wrong. These authors showed that the plasmids used for transforming *Serratia* were only transient and lost in vivo after three generations of mosquitoes, with the bacteria returning to the wild type [134] (Table 1). Also, concerns about the possible pathogenicity of any released transgenic bacteria towards animals and humans must be considered. For example, species/strains of insect endosymbionts used in paratransgenesis, such as *P. agglomerans*, *Asaia* and *Serratia*, have all been described as opportunistic pathogen in humans [203, 338, 339], although it is unlikely that these pathogens are the same as the bacteria isolated from insects. There are even reports of probiotics like *Lactobacilli* acting as human pathogens [340].

Nevertheless, it will be necessary to undertake risk assessment tests on the potential pathogenicity of transgenic bacteria of the sort described by Beard et al. [69], before their release into the environment [337]. Finally, the question of the target parasite potentially developing resistance to the effector molecules expressed by the transgenic bacteria has been addressed by Wang et al. [120], working with transformed *Serratia* AS1 in *An. stephensi.* The *Serratia* were engineered to produce, simultaneously, multiple effector genes with different targets in *Plasmodium falciparum*, significantly reducing the likelihood of possible resistance, and when the bacteria were fed to mosquitoes, 48 h before an infected blood meal, oocyst loads were reduced > 91% (Table 1).

While the above examples appear to answer many of the concerns of the regulatory bodies, they are insufficient by themselves to gain approval. First, semi-field experiments are limited in terms of number of insects utilised and subsequent risk assessments made of the effects on other insect species and the environment. Types of experimental data required by the European Food Safety Authority/European Commission (EFSA/EC) report on risk assessment [338] include: (i) demonstration of an exact understanding of the genetic modification of the GM organism; (ii) details of the release method and the receiving environment; (iii) any interactions (intended or unintended) between the GM organism and the recipient environment; and (iv) validated protocol details for monitoring and control of the GM organism following release [336]. Secondly, it is relatively early days in seeking approval for the use of paratransgenesis in the field and, as such, the structure of regulations governing the release of the engineered bacteria used may be insufficient.

The authors recommend consulting the EFSA document “Guidance on the environmental risk assessment of genetically modified animals” [337], which states clearly that “scientific activities in the area of GM animals indicate that future applications may include traits related to disease resistance” and “insects (e.g. mosquitoes, agricultural pests, bees)” are now part of the remit of this organisation. Other countries have their own bodies regulating the release of GM organisms; these include Brazil (the National Biosafety Technical Commission [CTNBio]) [341] and the USA (the Food and Drug Administration Center for Veterinary Medicine [FDA-CVM], the Centers for Disease Control and Prevention [CDC] and the Environmental Protection Agency [EPA]) [342]. Only now are some of these bodies developing regulations for the release of GM animals [341]. A search of the USA regulatory bodies (20 March 2021) identified only one reference to paratransgenesis, and this was for development of a paratransgenesis system to control Pierce’s disease of grapes (see EPA TSCA [343]). Contacting the relevant bodies above for guidance is highly recommended.

Anybody applying for approval for the release of transformed bacteria/vector insects in the environment should read the EPA documents submitted for field testing of genetically modified baculoviruses in the 1990s for the control of insect pests on plants [342]. Much of the work was concerned with the addition of scorpion toxin genes to enhance the kill rate of polyhedrosis viruses without a consequent change in host range of the viruses. These scorpion toxins modulate sodium channels but do not affect vertebrate activity, and testing with surrogate species and human cell lines also revealed no toxicity. It is, therefore, significant that scorpine has already been used successfully against *Plasmodium berghei* in *Anopheles gambiae* and *An. stephensi* mosquitoes in paratransgenisis experiments [e.g. 121]. However, regulations relevant to the 1990s would, no doubt, have been updated by now.

**Fig. 3:**
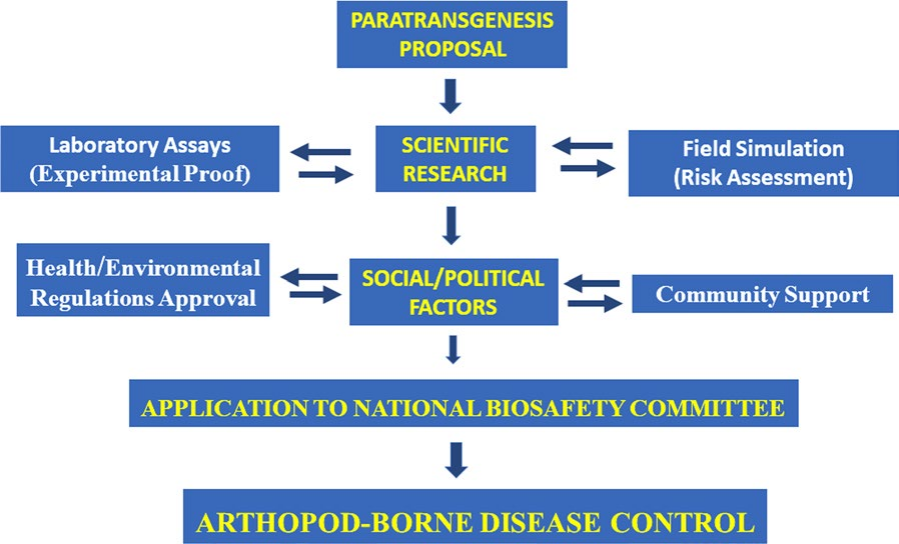
Some of the important considerations, apart from the laboratory and field experiments, which are vital for gaining approval prior to the release of genetically modified organisms, including mosquitoes, containing transgenic bacteria

Apart from the need for the appropriate scientific experimental work and satisfying the regulatory laws, there are important additional considerations before the GM organism can be released (Fig. 3). These include the social and public health aspects [344, 345], which were dealt with in detail during the field releases of Oxitec’s GM transgenic *Aedes aegypti* mosquitoes in the Cayman Islands, Brazil and Mexico [344–346]. The public health dimension provides evidence to justify the intervention of the GM organisms in a particular health risk. The failure of present control strategies to contain dengue infections would provide the need for such new strategies. The people actually involved in this process would include those at risk from dengue, scientists and regulatory bodies [344]. The social aspect is also very important as it involves the local community in the project and is essential for nurturing trust and approval for the GM mosquito release process [344, 345, 347]. Details for formulating these scientific, regulatory, public health and social dimensions are given in the references cited above. The whole of the GM release process can take years of collaborative work due to lack of any pipeline created by previous successful projects. Even with these dimensions fulfilled, there has recently been strong opposition to GM mosquito release in Florida despite EPA and CDC approval [347, 348].

Regarding the future of paratransgenesis, progress to approval is slow and the processes involved daunting. However, increases in pesticide and drug resistance and climate change have resulted in enhanced zoonoses and losses in food crops due to insect pests [349, 350], so that alternative strategies like paratransgenesis will be required in IPM schemes [351]. The development of paratransgenesis is one answer to the recent World Health Organisation’s “call for innovation” for “new malaria-fighting tools and approaches” [352]. The value of such new approaches will become self-evident once epidemiological results begin to show impacts on disease for the use of GM mosquitoes and paratransgenesis, and also indicate that the techniques could be cost-effective [346].

## Data Availability

All references cited are listed in the bibliography.

## Abbreviations

AgDNV: *Anopheles gambiae* densonucleosis viruses
AMP: Antimicrobial peptide
*Asaia*^GFP^: *Asaia* strain labelled with green fluorescent protein
BMI: Blood meal-induced promoters
CRISPRs: Clustered regularly interspaced short palindromic repeats
EFSA: European Food Safety Authority
EFSA/EC: EFSA/European Commission
EPA: Environmental Protection Agency
GFP: Green fluorescent protein
GM: Genetically modified
HTS: High-throughput sequencing
kanR: Kanamycin resistant
mCherry: Red fluorescent protein
MDVs: Mosquito densoviruses
PbGFPcon: *Plasmodium berghei* engineered to express GFP
pWCP: *Culex pipiens* plasmid
rDB3: Murine antibody fragments
rec*Sodalis*: Recombinant *Sodalis*
*RHBP*: *Rhodnius* heme-binding protein
SMI: Salivary gland and midgut molecules
