# Correction: Overview of paratransgenesis as a strategy to control pathogen transmission by insect vectors

**DOI:** 10.1186/s13071-024-06492-2

**Published:** 2024-09-27

**Authors:** Norman A. Ratcliffe, Joao P. Furtado Pacheco, Paul Dyson, Helena Carla Castro, Marcelo S. Gonzalez, Patricia Azambuja, Cicero B. Mello

**Affiliations:** 1 Programa de Pos‑Graduacao em Ciencias e Biotecnologia, Instituto de Biologia (EGB), Universidade Federal Fluminense (UFF), Niteroi, Brazil; 2 Department of Biosciences, Swansea University, Singleton Park, Swansea, UK; 3 Laboratorio de Biologia de Insetos, Instituto de Biologia (EGB), Universidade Federal Fluminense (UFF), Niteroi, Brazil; 4 Institute of Life Science, Medical School, Swansea University, Singleton Park, Swansea, UK

**Correction: Parasites & Vectors (2022) 15:112** 10.1186/s13071-021-05132-3

Following publication of the original article [1], it was pointed out that reference 325 was incorrect and linked to the wrong article. The correct reference is: Abassi R, Akhlaghi M, Oshaghi MA, Akhavan AA, Yaghoobi-Ershadi MR, et al. Dynamics and fitness cost of genetically engineered *Enterobacter cloacae* expressing defensin for paratransgenesis in *Phlebotomus papatasi*. J Bacteriol Parasitol. 2019;10:349. 10.4172/2155-9597.1000349.

The reference has since been corrected in the original article.
